# The genomic basis of environmental adaptation in house mice

**DOI:** 10.1371/journal.pgen.1007672

**Published:** 2018-09-24

**Authors:** Megan Phifer-Rixey, Ke Bi, Kathleen G. Ferris, Michael J. Sheehan, Dana Lin, Katya L. Mack, Sara M. Keeble, Taichi A. Suzuki, Jeffrey M. Good, Michael W. Nachman

**Affiliations:** 1 Department of Biology, Monmouth University, West Long Branch, New Jersey, United States of America; 2 Department of Integrative Biology and Museum of Vertebrate Zoology, University of California, Berkeley, Berkeley, California, United States of America; 3 Computational Genomics Resource Laboratory, California Institute for Quantitative Biosciences, University of California, Berkeley, Berkeley, California, United States of America; 4 Department of Neurobiology and Behavior, Cornell University, Ithaca, New York, United States of America; 5 Division of Biological Sciences, University of Montana, Missoula, Missoula, Montana, United States of America; 6 Department of Molecular and Computational Biology, University of Southern California, Los Angeles, Los Angeles, California, United States of America; University of Wisconsin–Madison, UNITED STATES

## Abstract

House mice (*Mus musculus*) arrived in the Americas only recently in association with European colonization (~400–600 generations), but have spread rapidly and show evidence of local adaptation. Here, we take advantage of this genetic model system to investigate the genomic basis of environmental adaptation in house mice. First, we documented clinal patterns of phenotypic variation in 50 wild-caught mice from a latitudinal transect in Eastern North America. Next, we found that progeny of mice from different latitudes, raised in a common laboratory environment, displayed differences in a number of complex traits related to fitness. Consistent with Bergmann’s rule, mice from higher latitudes were larger and fatter than mice from lower latitudes. They also built bigger nests and differed in aspects of blood chemistry related to metabolism. Then, combining exomic, genomic, and transcriptomic data, we identified specific candidate genes underlying adaptive variation. In particular, we defined a short list of genes with *cis*-eQTL that were identified as candidates in exomic and genomic analyses, all of which have known ties to phenotypes that vary among the studied populations. Thus, wild mice and the newly developed strains represent a valuable resource for future study of the links between genetic variation, phenotypic variation, and climate.

## Author summary

The recent introduction of house mice into North America from Europe provides an opportunity to investigate environmental adaptation in an important genetic model system. We found that mice from different latitudes differed in body size and aspects of blood chemistry and behavior, and that those differences have a genetic basis. Using exome and whole genome sequencing, we identified genes that show signals of selection. Gene expression in the laboratory also differed among mice from different latitudes. By combining approaches, we were able identify specific candidate genes for environmental adaptation. These results suggest wild mice may be a rich resource for future study of the genes underlying metabolic variation.

## Introduction

Understanding how organisms adapt to their environment is at the heart of evolutionary biology. The recent introduction of the western house mouse (*Mus musculus domesticus)* into North America from Europe provides a unique opportunity to study the genetic basis of environmental adaptation over short evolutionary timescales in the context of a genetic model system. While their time in the Americas may seem short, in most locations, mice breed seasonally and may produce two generations per year. Therefore, mouse populations have been evolving for ~400–600 generations in the Americas. In fact, some traits, including body size and nest building, are known to vary among populations and those differences have been shown to have a genetic basis [*e*.*g*., 1].

Connecting genotype, phenotype, and fitness remains challenging. Considerable progress has been made in uncovering the genetic basis of adaptation for traits that are controlled by one or a few genes of major effect, such as coat color in mice [e.g. 2, 3], ability to digest lactose in adult humans [4], or armor plating in sticklebacks [5]. However, adaptive evolution often involves traits controlled by many genes where gene-gene and gene-environment interactions are important. Less progress has been made in understanding the genetic basis of adaptive evolution for complex traits [but see 6, 7].

One approach to this problem is to conduct genome-wide scans for selection by looking at allele frequencies that co-vary with some aspect of the environment. Statistical methods have been developed that take population structure into account and thereby detect signals of selection above and beyond the patterns that are produced by the demographic history of the sampled populations [e.g. 8, 9]. Genome scans have now been applied to a wide range of organisms and have led to the identification of many candidate genes for adaptation [e.g. 10–18]. One strength of this method is that it is not predicated on phenotypes chosen *a priori*, and thus, in principle, might lead to the discovery of genes not previously suspected to underlie a particular adaptive phenotype [e.g. 19]. On the other hand, many studies using this approach produce a list of genes showing unusual allele frequency distributions, but fail to make connections between particular genes and either molecular or organismal phenotypes. Moreover, in cases where phenotypic differences are observed between wild populations, it is often unclear whether they reflect genetic differences or simply phenotypic plasticity in different environments. A genetic basis for phenotypic differences can be demonstrated by observing individuals from different populations in a common environment, as has been frequently done with plants [e.g. 20, 21]. In addition, gene expression provides an intermediate phenotype that can be used to connect genome scans to organismal phenotypes [e.g. 22]. Finally, a large body of literature on gene function can be used to link genetic and phenotypic variation in model species such as house mice.

Here, we use a combination of approaches to investigate the genomic basis of adaptation in house mice. First, we sampled mice across a latitudinal gradient ranging from Florida to Vermont, initiating lab strains from populations at the ends of the cline. By measuring traits in a common lab environment over multiple generations, we established that a number of complex traits related to fitness differ between populations and that those differences are genetically determined. Sequenced exomes and whole genomes of wild caught mice along the transect were used to identify genes showing signatures of selection. We then studied gene expression in lab progeny as an intermediate phenotype to highlight a set of genes likely connected to adaptive organism-level phenotypes. Mice serve as an important biomedical model, and the new inbred strains of mice developed here will be a valuable resource for phenotypic studies in the future.

## Results and discussion

### Phenotypic differences among populations

**Fig 1:**
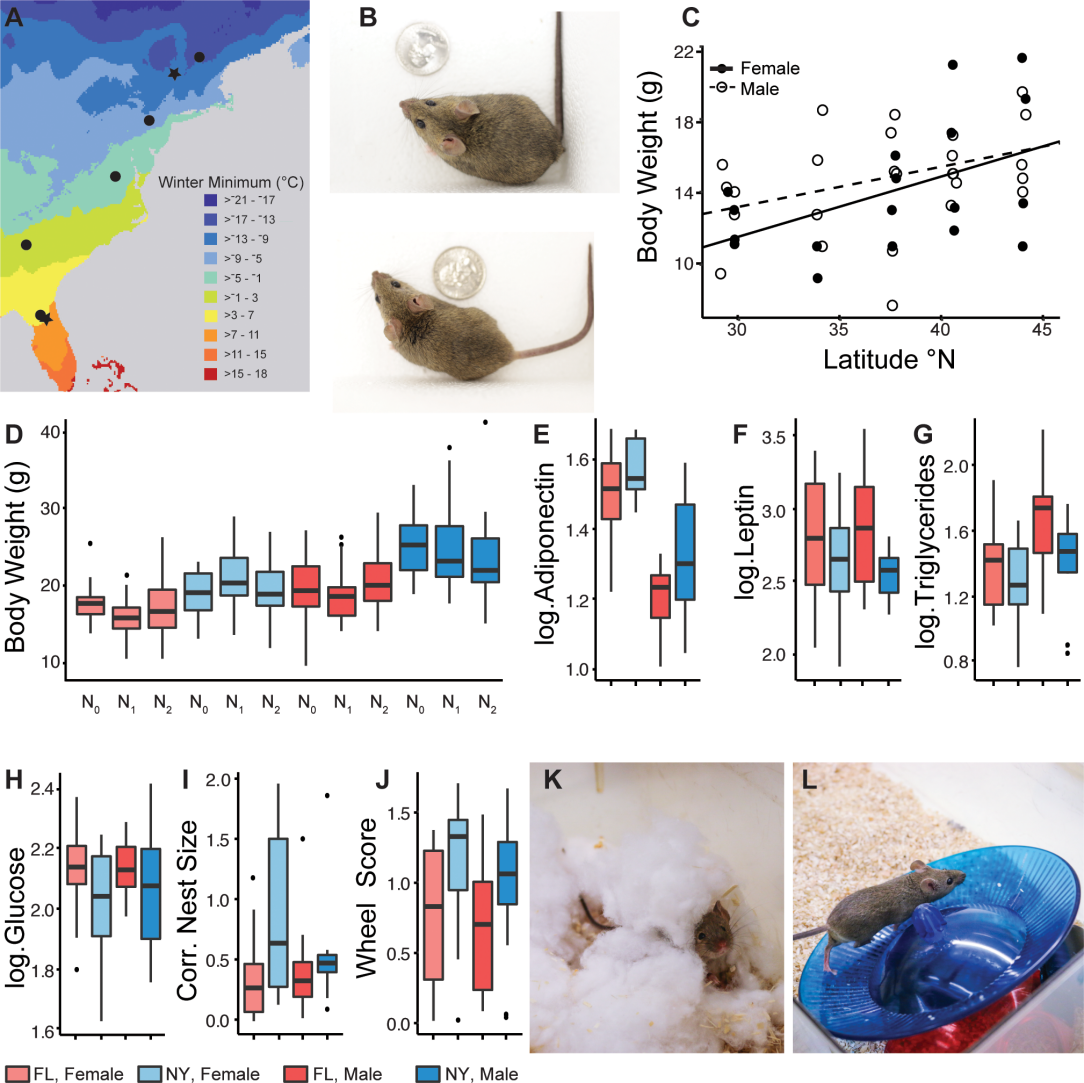
Phenotypic differences between mouse populations. **(A)** Winter minimum temperatures for the eastern US showing collection localities. Stars indicate live mouse collection localities **(B)** Lab-reared offspring of mice from New York (top; NY) are visibly larger than those from Florida (bottom; FL; photos by T. Suzuki). **(C)** Body weight in wild mice is linearly correlated with latitude (female: y = 0.332x + 1.592, n = 18, *r* = 0.492, *p* = 0.004; male: y = 0.214x + 6.54, n = 27, *r* = 0.379, *p* = 0.051; see methods for additional details). **(D)** Body weight differences among populations persist over two generations in the lab (*p* <0.0001; see methods for details). **(E-H)** N_2_ mice from NY show **(E)** higher levels of adiponectin (*p* = 0.046), **(F)** lower levels of leptin (*p* = 0.010), **(G)** lower levels of triglycerides (*p* = 0.024), and **(H)** lower levels of blood glucose (*p* = 0.028) than N_2_ mice from FL. **(I-L)** Behaviors also differ between populations. **(I)** NY N_2_ mice build larger nests (*p* = 0.003) and **(J)** are more active than FL N_2_ mice (*p* = 0.009). Example of **(K)** a nesting mouse and **(L)** a mouse running on a wheel (photos by G. Heyer).

We sampled ten wild house mice from each of five populations along a ~15° latitudinal gradient in Eastern North America over which major climatic factors vary linearly (Fig 1A). Each mouse was collected at least 500 m from every other mouse to avoid sampling relatives. This distance is well beyond the average dispersal distance of mice [23]. The sampled populations fall along a strong and predictable linear gradient in major climatic factors (Fig 1A; S1 Fig). Mice were sacrificed in the field, body measurements were recorded (total length, tail length, hind foot length, ear length, and body mass), tissues were collected for DNA sequencing, and museum specimens were prepared and have been deposited in the collections of the U.C. Berkeley Museum of Vertebrate Zoology (S1 Data). Mice from natural populations exhibited clinal variation in body weight, body length, and body mass index (BMI), with increasing body size in mice from colder environments (Fig 1B and 1C; S1 Table), consistent with Bergmann’s rule [24] and in accordance with earlier studies [1].

To determine whether population-specific phenotypic differences observed among wild mice are genetically determined or represent phenotypic plasticity, we collected live mice from the two ends of the transect (Saratoga Springs, NY and Gainesville, FL) and established laboratory colonies from wild-derived animals using brother-sister mating. We observed significant population-specific differences in body size measures of wild-caught, N_1_, and N_2_ mice across generations (Fig 1D; S2 and S3 Tables; S1 Data). We found that mice from New York (NY) were heavier, longer, and had higher BMI than mice from Florida (Fig 1D; S2 and S3 Tables). Differences in weight and BMI persisted into the second lab-born generation (N_2_) indicating that they mainly reflect genetic differences rather than phenotypic plasticity or maternal effects (Fig 1D; S3 and S4 Tables).

To better understand how these populations are adapted to their specific environments, we measured additional phenotypes in N_2_ lab-born progeny of mice collected from the ends of the transect (S1 Data). In particular, we reasoned that metabolic phenotypes might reflect adaptation to environments that differ in temperature and food availability for much of the year. We found that N_2_ mice from NY had significantly higher levels of adiponectin and lower levels of leptin, triglycerides, and glucose in their blood compared to mice from FL (Fig 1E and 1H; S3 and S5 Tables). These measures relate to glucose and lipid metabolism and are biomarkers for associated diseases in humans [25]. An inverse relationship between levels of adiponectin and the other measures is well established, but obesity is generally associated with lower adiponectin [25]. In this study, mice with higher BMI had higher levels of adiponectin. Interestingly, however, population-specific differences in adiponectin have been documented among healthy (non-obese) humans, with Europeans showing higher levels than people of African ancestry [26, 27]. The reasons for these differences remain unclear, but they mirror the latitudinal differences observed here in mice. Despite differences in body size, food intake did not differ among N_2_ mice from NY and FL (S3 and S6 Tables). We also measured nest building and wheel running in N_2_ mice. Nest building has clear links to fitness via effects on thermoregulation in neonates [28] and there is evidence that wheel running is associated with metabolic rate in rodents [29]. We found that mice from NY built bigger nests than those from FL (Fig 1I and 1K; S3 and S7 Tables), consistent with earlier studies [1], and had higher activity levels (Fig 1J and 1L; S3 and S8 Tables).

### The genomic signature of environmental adaptation

To identify the genetic basis of these differences, we sequenced the complete exomes of the 50 wild-caught mice at moderate coverage (S9 Table). We used several different approaches to identify candidate genes underlying environmental adaptation (see Methods for a detailed description of the methods and the rationale for each). To account for the confounding effects of population structure which may arise from the demographic history of populations, we used a Latent Factor Mixed Model (LFMM), a program that implements a variant of Bayesian principal component analysis in which neutral population structure and covariance between environmental and genetic variation are simultaneously inferred [9]. LFMM outliers were identified using a z-score cut-off and a False Discovery Rate (FDR) correction (see Methods). However, in these data, there was no significant evidence for isolation-by-distance among populations (S10 Table;S2 Fig). Neighboring populations were not more closely related to each other than were more distant populations. While the demographic history of these populations is not explicitly modeled here, the lack of correlation between patterns of genetic differentiation across the genome with geographic distance suggests an alternative approach to detecting selection—identifying loci that show clinal variation [30]. We therefore also used linear regression to identify SNPs at which allele frequencies vary clinally with latitude. Latitude was used as a proxy for climatic variation due to its strong correlation with the first principal component summarizing climatic variables (Pearson’s r = -0.99, df = 3, *p* < 0.0006). We identified two classes of SNPs. The first included SNPs that were in the top 5% of the distribution for R^2^ and in the top 5% of the distribution for the absolute value of slope even when any one population was dropped from the analysis (S3 Fig). The second included SNPs that were in the top 2.5% of the distribution for R^2^, regardless of the slope, even when any one population was dropped from the analysis (S3 Fig). While clinal patterns with large differences in allele frequency are consistent with strong selection, clinal patterns with more subtle differences in allele frequency are expected in a number of scenarios including selection on standing variation and on complex traits [31]. After FDR correction, the outliers for both classes of SNPs were significant (*q* < 0.01; see Methods).

**Fig 2:**
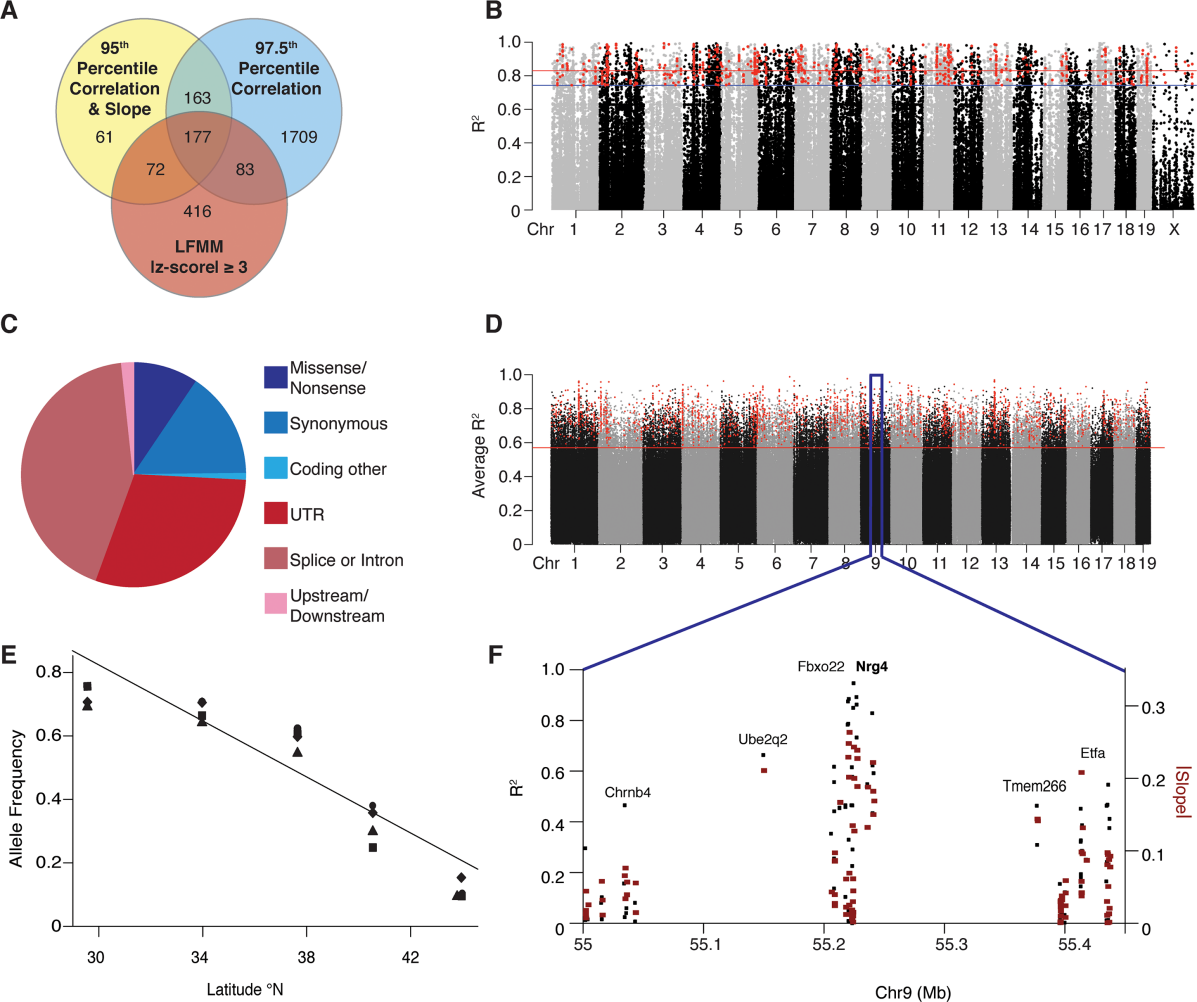
Candidate genes. **(A)** Overlap between genes with candidate SNPs identified using different methods in the exome: linear regression with a 95% cutoff for R^2^ and |slope|, linear regression with a 97.5% cutoff for R^2^ alone, and LFMM |z-score| ≥ 3. **(B)** The minimum R^2^ for the linear relationship between allele frequency and latitude for each SNP in the exome when including all populations or dropping any one population. Red and blue lines mark the top 2.5% and 5% respectively of the distribution for R^2^ when including all populations; SNPs that also have a |slope| in the top 5% are highlighted in red. **(C)** The distribution of SNPs among annotation categories for candidates identified in the exome using a regression approach with a 95% cutoff for R^2^ and |slope|. **(D)** Average R^2^ for the linear relationship between allele frequency and latitude for 2.5 kb non-overlapping windows in the genome. The red line marks the top 5% of the distribution for R^2^; windows that also have a |slope| in the top 5% are highlighted in red. **(E)** Four SNPs in *Fbxo22*/*Nrg4* are highly correlated with latitude with steep shifts in allele frequency (one regression line shown for clarity). **(F)** Exome data show that the signal of selection drops off around *Fbxo22/Nrg4*.

Each method identified several hundred loci containing outlier SNPs (S1 Data). There was significant overlap among the sets of loci identified using the different methods (permutation test, *p* < 0.0001; Fig 2A). Candidates were distributed throughout the genome (Fig 2B). It is not possible to precisely delineate the decay of linkage disequilibrium with discontinuous exomic data. However, signals generally did not extend over large chromosomal distances. For example, in > 70% of the genes identified by all three cut-offs, elevated LFMM scores extend less than 25kb upstream and downstream, consistent with estimates of the decay distance of linkage disequilibrium in mouse populations (Fig 2B; S11 Table; [32]). This pattern is also consistent with selection on standing variation [33] and suggests that the results provide resolution to individual genes in most cases. Classical strains of laboratory mice provide a rich catalog of allelic variants, including loss-of-function alleles that have been associated with specific phenotypes [MGI: MouseMine; 34,35]. Phenotypes known to be associated with the genes identified here include many of those observed to be different between mice from Florida and mice from New York, such as body weight, body fat, activity level, behavior, glucose metabolism, and leptin and adiponectin levels.

Less than 10% of clinal SNPs in the exome-capture dataset were annotated as non-synonymous or missense mutations (Fig 2C), roughly equivalent with the fraction of variable sites that were classified as non-synonymous or missense sites (9.5% and 9.2%, respectively; S12 Table). Most clinal SNPs were in introns (~42%), UTRs (~30%), or at synonymous sites (~15%); these SNPs (if true positives) may either underlie environmental adaptation or be in linkage disequilibrium with causative SNPs. Importantly, <15% of protein-coding genes identified as candidates in the regression analyses contain a non-synonymous or missense outlier SNP. Results were similar for candidates identified using LFMM (S12 Table). While there is no enrichment for regulatory regions, these results suggest that changes in gene regulation contribute to adaptation in this system.

To further explore the signal of regulatory evolution, we sequenced, at low coverage, the complete genomes of the same 50 wild-caught mice included in the exome analysis (S13 Table). Candidate regions underlying adaptive differences were identified using sliding windows of average R^2^ and |slope| from linear regressions of allele frequencies of SNPs in the genomic data with latitude. Given the low coverage, most sites could not be called for all individuals and data were insufficient for analysis of the X chromosome. Despite this, estimates of allele frequencies in the autosomal data were highly correlated with estimates from the exome data in the entire dataset (Pearson’s r = 0.97, df = 242,136, *p* < 3 x 10^−16^; S4A Fig) as well as within individual populations (Pearson’s r = 0.90, df = 989,907, *p* < 3 x 10^−16^; S4B Fig). Candidate regions were distributed throughout the genome (e.g., Fig 2D). Interestingly, approximately half of all these regions fell within 5 kb of a gene, suggesting that while many candidate regions lie in or near genes, many do not (S14 Table). Approximately 10% of candidate windows were within ± 500 bp of a putative promoter [Mouse ENCODE; 36], ~75% of which also fell within 5 kb of a gene (S14 Table). The genes identified in this analysis overlapped significantly with the genes identified using the exome-capture data (permutation test, *p* < 0.0001).

### Differences in gene expression between populations and identification of *cis*-eQTL

Because changes in gene regulation appear to contribute to adaptive evolution in this system (Fig 2C), we measured differences in gene expression between lab-born progeny of wild-caught mice from the ends of the transect. In principle, patterns of gene expression can be used to make connections between genotype and organismal phenotypes. Many of the observed phenotypic differences between mice from the ends of the transect are related to metabolism (Fig 1), thus we measured gene expression in tissue from the liver, adipocytes from fat pads on the hind limb, and the hypothalamus using RNAseq. Expression was measured in unrelated adult N_1_ progeny reared in a common environment and matched for age and sex. To address potential maternal effects, liver tissue from unrelated adult N_2_ males was also included. Principal components analysis (PCA) clearly distinguished the two populations in all tissues, including liver from second-generation lab-born mice (S5 Fig). The persistence of differences into the second generation in the lab suggests that observed differences in gene expression are not likely to be mainly due to maternal effects. PCA was also used to identify outliers that were removed from further analysis (S6 Fig). Differentially expressed genes were observed in all tissues, with fat showing the greatest number by far, suggestive of the potential biological significance of observed differences in metabolism and morphology (S15 Table).

Differences in gene expression may be caused by mutations in *trans* or by mutations in *cis*. The genomic locations of *trans*-acting mutations are difficult to identify, however *cis*-acting expression quantitative trait loci (*cis*-eQTL) may be identified by measuring allele-specific expression patterns in heterozygous animals [e.g.^37^–^39^]. If expression differences between mice from the ends of the transect reflect adaptation to different environments, we reasoned that a subset of genes harboring *cis*-eQTL might overlap with those showing signatures of selection in the exome or whole-genome analysis, allowing us to identify candidate loci with strong evidence of local regulatory variation. Allele-specific patterns of expression in heterozygous mice identified *cis*-eQTL in >3,500 genes across all tissues (S15 Table).

### Candidate genes for environmental adaptation

The different datasets and analyses presented here each identify sets of candidate genes that may underlie environmental adaptation in mice. One challenge of outlier approaches and genome scans more broadly is sifting through the false positives to identify true signals of selection. Here, we focused on candidates identified by LFMM for which there was also evidence of clinal patterns of allele frequency and large shifts in allele frequency. Then, in order to forge stronger links between genomic outliers and variation in traits related to fitness, we searched for overlap between those genes and genes showing expression differences between populations and genes harboring *cis*-eQTL. Specifically, 177 genes were identified at the intersection of the methods used in the analysis of exome sequences (Fig 2A). Of these, 127 were also identified in the window analyses of the low-coverage whole-genome data, and of these, 10 showed significantly different levels of expression in the lab and also were associated with a *cis*-eQTL (Table 1). When comparing the two most extreme populations, the distribution of estimated *F*_*st*_ values for candidate genes was skewed compared to the full list of genes (S7 Fig). Average per gene estimates of *F*_*st*_ for candidate genes were significantly higher than that of the full list of genes for which *F*_*st*_ could be estimated (full list: $\bar{F_{st}}$ = 0.103, sd = 0.105, n = 20,366; 177 candidates: $\bar{F_{st}}$ = 0.268, sd = 0.109, n = 162, t = 19.06, df = 163.38, *p* < 2.2 x 10^−16^; 127 candidates: $\bar{F_{st}}$ = 0.266, sd = 0.095, n = 122, t = 18.88, df = 122.79, *p* < 2.2 x 10^−16^; 10 candidates: $\bar{F_{st}}$ = 0.227, sd = 0.065, n = 10; t = 6.06, df = 9.02, *p* < 0.0002). In two-sided, two-sample Kolmogorov-Smirnov tests implemented in R, results indicate that the *F*_*st*_ estimates for the full set of genes and for each set of candidates in turn (177, 127, and 10 genes) do not come from the same distribution (*p* < 2.2 x 10^−16^, p < 2.2 x 10^−16^, *p* < 4.164 x 10^−5^, respectively). Results this extreme were not observed in permutation tests for each set (1000 replicates with replacement).

**Table 1:** Genes identified as candidates from all analyses in the exome and in the genome that show evidence of differential expression (DE) and allele specific expression (ASE) in comparisons between lab-bred mice from derived from populations in Florida and New York. Tissues come from N_1_ mice unless noted (F = Fat, L = Liver, H = Hypothalamus).

				Function, Phenotype, or QTL related to:			
Gene Name	Chr:Start(bp)	Tissue w/ evidence of DE	Tissue w/ evidence of ASE	Body Size/Fat/ Obesity	Circadian Rhythm	Immunity	Blood Chemistry/ Diabetes
*Col3a1*	1:45,311,538	F	F	Y	N	Y	N
*Rgs16*	1:153,740,349	F	L N_2_	Y	Y	Y	Y
*Mvb12b*	2:33,729,953	F	H	Y	N	Y	Y
*Pomp*	5:147,860461	F	F, H, L N_1_ & N_2_	N	N	N	Y
*Ndufa4*	6:11,900,292	F	L	Y	N	N	N
*Fbxo22*	9:55,208,925	F	F, L	Y	N	Y	N
*Nrg4*	9:55,220,222	F	F	Y	N	Y	Y
*Tmie*	9:11,0865,711	L N_1_ & N_2_	H	Y	N	Y	N
*Gpr108*	17:57,234,914	F	L	Y	N	Y	N
*Dsc2*	18:20,030,633	F	L N_2_	Y	N	N	Y

All of the 10 genes identified at the intersection of genome scans and gene expression studies (Table 1) are known to be associated with phenotypes that distinguish mice from the ends of the transect. For example, we identified two overlapping candidate genes on chromosome 9, *Fbxo22* and *Nrg4* (Fig 2E and 2F). While less is known regarding *Fbxo22*, *Nrg4* is highly expressed in adipose tissue and is linked to obesity and diet-induced insulin resistance in mice and humans [40, 41]. In obese mice, *Nrg4* appears to exert a beneficial effect, reducing the effects of metabolic disorders associated with obesity [40, 42, 43]. In human studies of obese adults, concentrations of *Nrg4* are inversely correlated with risk of metabolic syndrome [43].

Since regulatory regions are sometimes located far from genes, we were also interested in identifying those loci that showed signatures of selection in the whole genome data (but not necessarily in the exome data) and for which there was evidence of differential expression and allele specific expression in the same tissue for lab-bred mice derived from populations in Florida and New York. These criteria identified 40 additional genes (S16 Table). The distribution of *F*_*st*_ values for these genes, comparing the two most extreme populations, was also skewed compared to the distribution for the full list of genes for which *F*_*st*_ could be estimated (S7 Fig; two-sided, two-sample Kolmogorov-Smirnov tests, *p* < 5.2 x 10^−7^). Results this extreme were not observed in permutation tests (1000 replicates with replacement). The average per gene estimates of *F*_*st*_ for these candidate genes was significantly higher than that of the full list of genes for which *F*_*st*_ could be estimated (full list: $\bar{F_{st}}$ = 0.103, sd = 0.105; 43 candidates: $\bar{F_{st}}$ = 0.191, sd = 0.100, n = 41, t = 5.64, df = 40.18, *p* < 1.5 x 10^−6^). Most of these 40 genes are linked to phenotypes that differ between mice from the ends of the transect. *Cav1*, for example, affects the regulation of fatty acids and cholesterol [e.g. 44]. Knockout mice show reduced adiposity and resistance to diet-induced obesity. *Cav1* overlaps with QTL related to body size/growth and was identified as a candidate gene for extreme body size in Gough Island mice [45–47]. Gene network analyses in humans identify CAV1 as a key driver of cardiovascular disease and type 2 diabetes [48].

It is important to recognize that the different datasets analyzed here contain distinct kinds of information, and overlap is not expected in many cases. Therefore, while overlap among the results points to candidates, many candidate genes that contribute to environmental adaptation likely do not meet all criteria. For example, data on gene expression is limited by the tissues and time points considered. Therefore, some candidate genes may not show expression differences and/or may not harbor *cis*-eQTL, yet these genes may still be important in adaptive phenotypic differences. For example, multiple SNPs in *Mc3r* are strongly clinal, with some of the highest shifts in allele frequency seen in the exome (S8 Fig). Lab mouse variants at *Mc3r* are associated with leptin levels, energy homeostasis, body fat, and activity levels [49–54]. *Mc3r* is expressed in the hypothalamus [55], but levels of expression observed in this study were low and no significant differences were detected. Moreover, the phenotypes measured here do not include all that might be important in environmental adaptation. Some of the candidate genes that do not relate to the phenotypes directly measured here do relate to other phenotypes that may underlie environmental adaptation such as immunity and circadian rhythm, motivating future functional studies.

### Phenotypic and genetic parallels between mice and humans

Importantly, these results suggest that understanding environmental adaptation in mice may shed light on human disease and climate-related adaptation in humans. The phenotypic variation in mouse populations observed here over a latitudinal cline parallels differences in human populations. Humans, like mice, follow Bergmann’s rule, with larger individuals at higher latitudes [56–58]. Further, while the relationship between adiponectin, leptin, trigyceride, and glucose levels and obesity in humans is complex, the pattern of differences in these mouse populations is similar to that observed between some human populations [26,27]. Moreover, many of the phenotypes that vary and the candidate genes identified in the overlap analyses have ties to metabolic diseases and/or blood chemistry variables associated with these diseases. There are already mouse models for diseases like diabetes, but laboratory strains lack much of the genetic variation present in wild mice [59]. Interestingly, there is overlap between the genes identified here and those implicated in climate-related adaptation in humans in a series of studies by DiRienzo and colleagues [60–62]. Of the genes they identified, 43 have one-to-one orthologs in mice, and 18 of these were identified as showing signatures of selection in the LFMM analyses of the exome data (|z-score| ≥ 2; S17 Table). Moreover, nine also showed evidence of allelic imbalance, differential expression, or both. While this result is, at most, suggestive, the overlap between the genes identified here and those identified in humans is slightly more than expected by chance (permutation test, p<0.03), pointing to some commonality to the genetic basis of environmental adaptation despite different geographic sampling and ~ 90 million years of divergence between humans and mice [e.g., 63–65].

### Conclusions

Using an integrative approach, we were able to make connections between genetic and phenotypic variation for complex traits related to fitness. We found strong evidence of environmental adaptation in house mice. Wild mice show clinal patterns of variation in body size. Lab-born progeny of wild mice from different environments differ in body size, metabolic traits, and behavioral traits, indicating that these differences are genetically based. Genome scans for selection revealed that most candidate SNPs likely affect gene regulation. We identified a short list of genes that show signatures of selection, are associated with a *cis*-eQTL, exhibit differential expression, and are associated with organismal phenotypes in laboratory mice similar to the phenotypic differences seen in mice from the ends of the transect. These results underscore the value of investigating wild variation in a genetic model system. Future studies surveying more individuals within sites and more sites across a broader landscape would increase the power to detect allele frequency shifts consistent with environmental adaptation, allow for investigation of site-specific local adaptation, and provide a clearer picture of the colonization and demographic history of house mice in North America. The resources developed here, including new wild-derived inbred strains of mice and extensive exomic and genomic data, will facilitate future research aimed at uncovering the genetic basis of adaptation as well as broader studies investigating genetic and phenotypic variation in house mice.

## Methods

### Ethics statement

This work was conducted with approval from the IACUC of the University of Arizona (Protocol #07–004) and the IACUC of the University of California, Berkeley (Protocol #R361-0514, AUP-2016-03-8548). All wild-caught animals were collected with permits issued from the states of Florida, Georgia, Virginia, Pennsylvania, New Hampshire, New York, and Vermont.

Sacrifice of animals was also performed under approval of the relevant IACUC either at the University of Arizona or the University of California, Berkeley. Methods of euthanasia included the humane use of isoflurane and cervical dislocation by trained personnel.

### Collections

Five sampling locations were selected along a latitudinal gradient over which many climatic factors covary (Fig 1A, S1 Fig). At each location, at least ten individuals were collected a minimum of 500m apart to avoid collecting closely related animals. While larger sample sizes would increase the power to detect smaller differences in allele frequencies among populations, previous studies suggest that the sample sizes employed here are sufficient [e.g. 66]. Sex, reproductive status, body weight, total body length, tail length, hind foot length and ear length were recorded for each mouse along with latitude and longitude and elevation (S1 Data). Weight and length were measured in the field by a single investigator using a micro-line spring scale and a ruler. Animals were collected and sacrificed in accordance with a protocol approved by the Institutional Animal Care and Use Committee (IACUC) of the University of Arizona. Liver, kidney, heart, caecum, and spleen were collected in the field, stored on dry ice, and then transferred to a ^-^80°C freezer. Skins, skulls, and skeletons were prepared as museum specimens and deposited in the Museum of Vertebrate Zoology, University of California, Berkeley (see S1 Data for accession numbers).

To characterize climate for each location on the transect, data for all BioClim variables from the WorldClim database [67] were downloaded with a resolution of 2.5 arc-minutes using the R package dismo using coordinates roughly central to all individual collection sites within each location (S1 Data). Additional data were also downloaded from the National Centers for Environmental Prediction/National Center for Atmospheric Research (NCEP/NCAR) Reanalysis 1 using the R package RNCEP [68]. These variables include net shortwave radiation, specific humidity, relative humidity and sunshine hours. Because many climatic variables co-vary, climate data were standardized and then summarized using principal components analysis (PCA) on correlations including all variables (S18 Table). The first PC explained ~71% of the variation among populations, and almost all of the variables had large loading values for this axis. Latitude is highly correlated with PC1 (R^2^ = 0.98, df = 3, *p* < 0.001).

Live animals were collected from two locations, Saratoga Springs, NY and Gainesville, FL (Fig 1A). Within locations, no more than two breeding pairs from a single site were included, and sites were no closer than 500 m from each other. Animals were collected and shipped alive to the University of California, Berkeley, where they were used to establish colonies. All work was performed in accordance with a protocol approved by the Animal Care and Use Committee (ACUC) of U.C. Berkeley. Wild-caught animals were mated to create the first lab-reared (N_1_) generation (S1 Data). N_2_ mice were generated via brother-sister mating of the N_1_ mice. Inbred lines have subsequently been maintained via sib-sib matings. We currently have 8 lines from each of these two locations, and most lines are in the tenth or later generation of sib-sib mating.

### Phenotyping and analyses: Field

Analyses of the correlation between latitude and measures of body size were completed using R and included all animals, only females, and only males, respectively. Pregnant and/or lactating females and one juvenile male were excluded from the analyses. There was a significant correlation between latitude and body mass from field collections along the transect when both sexes were included (Fig 1B and 1C; S1 Table). Clinal variation was also seen in body length, body mass corrected for length, and body mass index (BMI) (S1 Table). When considering the sexes separately, only body weight and body mass/body length were significantly correlated with latitude in females (S1 Table; *p* = 0.004, *p* = 0.004, respectively), but correlations in males were marginally significant (*p* = 0.051, *p* = 0.054, respectively) and trends were clinal for all traits in both sexes.

### Phenotyping and analyses: Laboratory

Experimental mice phenotyped in the lab were housed singly in standard static cages at 23°C with 10 hour dark and 14 hour light cycles. Body weight and body length were measured for over 300 wild-caught, N_1_, and N_2_ mice (see S1 Data) and Body Mass Index (BMI) was calculated from those measures. In total, we obtained data for 49 wild-caught, 56 N_1_, and 84 N_2_ mice from FL and 21 wild-caught, 77 N_1_, and 63 N_2_ mice from NY. To test whether body mass was significantly different between lab reared mice from Florida and New York, we used a generalized linear model (GLM) implemented in R including all mice with generation, population, and sex as factors to explain body mass (S2 and S3 Tables). Results were evaluated using the anova function; F test results are reported (S2 Table), but the choice of test type does not affect whether individual factors meet the criteria for significance. We repeated the analysis for body length, BMI, and body mass divided by body length (S2 and S3 Tables). We also analyzed the data from just the N_2_ generation using GLMs with population, sex, and age as predictors of each aspect of body size (S3 and S4 Tables).

A subset of the N_2_ mice was also included in phenotyping for blood chemistry, food intake, nesting, and wheel running. For blood chemistry measurements, 20 mice from FL and 20 mice from NY were sacrificed at an average age of 26.68 weeks (sd = 2.63) between 1-5pm after fasting for 2–7 hours. There was no significant difference in age between the mice from FL and the mice from NY (${\bar{age}}_{FL}$ = 26.55, sd_FL_ = 3.74; ${\bar{age}}_{NY}$ = 26.80, sd = 1.47; t = 0.30, *p* = 0.79). 100–500 μl of blood was extracted from the heart and body cavity using a syringe and 22-gauge needle. Serum was isolated using BD Microtainer tubes with a serum separator additive. To measure potential differences in metabolism related to blood chemistry, standard assays of insulin, leptin, adiponectin, glucose, trigylcerides, free fatty acids, cholesterol, and HDL were performed at the UC Davis Mouse Metabolic Phenotyping Center. To test for significant differences in blood chemistry between lab reared N_2_ mice from New York and Florida we used separate linear mixed effects models for each measure with population, sex, log(mass) and log(length) as factors taking into account family (S5 Table).

Food intake, nest building and wheel running activity were observed in the N_2_ mice at an average age of 12.97 (sd = 2.63), 15.28 (sd = 2.64), and 25.55 (sd = 7.54) weeks, respectively (S1 Data). Daily food intake was measured by administering 35g of Teklad Global food (18% Protein Rodent Diet) to each animal, and then weighing the remainder 24 hours later. All mice were fed *ad libitum* prior to testing. Nest building behavior was assayed by placing 40g of cotton on top of the wire of each cage and weighing the remaining unused cotton 24 hours later. To determine if either food intake or nest building behavior was significantly different between lab-reared mice from Florida and New York, we used separate GLMs with population, sex, and body mass as factors (S6 and S7 Tables). Wheel-running activity was assayed by attaching a Speedzone Sport Wireless bike odometer (Specialized) to a Fast-Trac Activity Wheel (Bio-Serv). Running trials began at the start of the dark cycle (9:00 pm), and running distance and time spent running were recorded at the end of the dark cycle. Distance was corrected for slight differences in run time and was log transformed. A GLM with population and sex as factors was used to determine if there were differences in wheel-running activity between mice from NY and FL (S8 Table). All mice that did not run at all, including two mice from NY and 6 from FL, were excluded from the analysis. The average, standard deviation, and sample size by population for each measure in the analyses above are given in S3 Table.

### Exome capture sequencing and SNP discovery

DNA was extracted from liver, kidney or spleen tissue using the Qiagen Gentra Puregene Kit. Genomic libraries were prepared following Meyer and Kircher [69] with unique barcodes added for each individual. A NimbleGen in-solution capture array was used to enrich the libraries for regions in the mouse exome (SeqCap EZ). Targeted areas include ~ 54.3 Mb of nuclear coding and UTR sequence. Individuals were pooled for capture in groups of sixteen or seventeen. Each pool of enriched capture libraries was then sequenced on one lane of a Illumina HiSeq2000 (100-bp paired-end) resulting in ~2 GB of raw data per individual.

Sequence data were cleaned using a combination of custom perl scripts and publicly available programs as in Singhal [70; see also https://github.com/CGRL-QB3-UCBerkeley/denovoTargetCapturePopGen]. These scripts remove adapter sequences, filter out low complexity reads, bacterial contamination and PCR duplicates, and merge overlapping paired reads. The cleaned reads were then mapped to the mouse genome (GRGm38) using Bowtie 2.1.0 [71] using the sensitive setting, trimming three bases from both the 3’ and 5’ ends of each read, and allowing no discordant mapping for paired reads. Reads that did not map or that mapped to multiple regions were removed, and target specificity and sensitivity were evaluated (S9 Table). On average, ~63% of the data for an individual mapped to target regions and ~92% of the targeted exome was covered. Overall, average sequence depth per site was ~15X. Data from the Y chromosome was used to estimate error rates based on heterozygote calls for males included in the study (average = 0.026%, sd = 0.004%, n = 25).

Individual sites were additionally filtered using a custom perl program, SNPcleaner [72], with default parameters with the exception of requiring 3X coverage in at least 80% of the individuals. We called SNPs and estimated allele frequencies at variable sites using the software ANGSD [73], a package that uses a Bayesian framework to address biases that result from calling variant sites and genotypes with low to moderate coverage sequence data [74]. To be included in further analyses, the posterior probability for the genotype of the individuals had to be ≥ 0.50 and the *p*-value of the likelihood ratio test for a SNP being variable had to be ≤ 0.001. These filters resulted in the identification of ~420,000 SNPs throughout the exome. Because subsequent analyses depended on an assessment of the shift in allele frequencies over a latitudinal gradient, we further required that there were data for eight individuals from each of the five sampled locations. This additional filter reduced the number of SNPs to ~408,000. Finally, we required that the minor allele frequency of a SNP across all individuals be at least 5%, resulting in a total of ~280,000 SNPs.

### Genome sequencing and SNP discovery

DNA was extracted from liver, kidney or spleen tissue using the Qiagen Gentra Puregene Kit. Genomic libraries were prepared using Illumina Truseq kits with unique barcodes added for each individual. Libraries from two or three individuals were sequenced on one lane of a Illumina HiSeq2000 (100-bp paired-end) at the Vincent J. Coates Genomics Sequencing Laboratory at UC Berkeley resulting ~9–19 GB of raw data per individual.

As with the exome data, genomic sequence data were cleaned using a combination of custom perl scripts and publicly available programs as in Singhal [70]. However, because of the additional computational time required to process low coverage, whole genome data, we did not remove PCR duplicates before mapping. After cleaning, reads were mapped to the mouse genome (GRCm38) using the sensitive setting, trimming three bases from the 3’ and 5’ ends of reads, and using the option to disable alignment of paired reads as unpaired. Unmapped and multiply mapped reads were then removed and Picard [https://broadinstitute.github.io/picard/] was used to remove PCR duplicates. Error rates for individuals were evaluated using mtDNA sequence data. The average error rate was generally low (average = 0.062%, sd = 0.024%, n = 49) with the exception of a single individual with an error rate of 0.29%. Average coverage of the total genome across individuals was ~2.5X. Average coverage for the sites at which each individual had at least one read mapped was slightly higher, ~3.3X (S13 Table).

All sites for which 80% of the individuals had data were included in subsequent population genetic analyses (e.g. *F*_*st*_ and PCA). However, for all analyses of variant sites, we used ANGSD to call SNPs and estimate allele frequencies for populations. We first applied a liberal filter, only estimating allele frequencies for those sites that had a posterior probability for the genotype of included individuals ≥ 0.50 and a *p*-value of the likelihood ratio test for that SNP being variable ≤ 0.001. As with the exome data, we further required that there were data for eight individuals from each of the five sampled populations and that the minor allele frequency of a SNP across all individuals be at least 5%, resulting in a total of ~9,800,000 SNPs.

Low coverage, whole genome data has the potential to identify variants associated with environmental adaptation far from genic regions at low cost. However, the utility of such an approach is dependent on the reliable identification of candidate variants with very little data for a single individual. To test this approach, we calculated the correlation coefficient between allele frequency estimates based on moderate coverage data from the exome and those based on low coverage data from the genome. We restricted the data to those sites in common and used the filtering described above. We found a high correlation between the allele frequency estimates from the two approaches given the entire pool of fifty individuals (Pearson’s r = 0.97, df = 242,136, *p* < 3 x 10^−16^; S4A Fig). We also found a high correlation between allele frequencies estimates within the individual populations of just ten individuals (Pearson’s r = 0.90, df = 989,907, *p* < 3 x 10^−16^; S4B Fig).

### Population genetic analyses

Data from the exome and the genome were used, in turn, to estimate *F*_*st*_ a measure of differentiation among populations using the unfolded site frequency spectra (SFS) generated for each population via ANGSD (e.g. *F*_*st*;_ S10 Table). We also used genetic PCA to summarize variation within and among populations. Both *F*_*st*_ calculations and genetic principal component analyses were implemented via the ngsTools software package [75]. Estimates of *F*_*st*_ varied among population pairs (S10 Table). Genetic PCA clearly discriminated populations, and *F*_*st*_ values provide evidence of population differentiation. Importantly, however, there was no significant signal of isolation by distance (S10 Table; S2 Fig). Statistical analyses including Mantel tests and reduced major axis regression were completed as given in [76]. While individuals were most closely related to other individuals from their own sampling location, there was no association between geographic and genetic distance among populations regardless of the data used (genomic or exomic; S2 Fig). *F*_*st*_ was also estimated for each gene using the exomic data. Genomic coordinates (5’ UTR-3’UTR) were obtained using Ensembl Biomart. Sites were only included when at least 80% of the samples had at least 3X coverage. Two-sample, two-sided Kolomogorov-Smirnov tests implemented in R were used to test whether the *F*_*st*_ estimates for the full set of genes (20,367) and for different sets of candidate genes were drawn from the same distribution (S7 Fig). Permutation tests with 1,000 replicates, also implemented in R, were used to determine how many such results were expected when the same number of genes were drawn, with replacement, from the full gene list. The significance of the differences between the means of the distributions was determined via t-tests also implemented in R.

ANGSD was also used to estimate nucleotide diversity within populations. Coordinates for all intronic sites and for all gene boundaries were obtained using Ensembl’s Biomart tool. Intronic sites from the exome data were then used to estimate Watterson’s θ and π. For the genomic data, average per site Watterson’s θ and π were estimated for 10kb non-overlapping sliding windows. Windows that overlapped with any portion of a gene were excluded (S19 Table). Overall, estimates of nucleotide diversity were high and comparable to estimates from European populations of *Mus musculus domesticus* [77]: genomic windows: 0.1694–0.2963; intronic sites: 0.1388–0.2332.

### Identifying candidate regions contributing to environmental adaptation in the exome

There are several approaches to identifying candidate genes under selection using genome scans, and each has advantages and limitations. One approach is to model the demographic history of a population, usually conditioned on some summary of available polymorphism data, and then to compare the observed data with model predictions. Individual loci that do not fit the model are inferred to have been subject to selection [e.g. 78, 79]. A limitation of this method is that it requires the correct specification of population history, which in practice is unknown. Incorrect model specification can lead to either false positives or false negatives. In recognition of this, a second widely adopted approach is to generate an empirical distribution of a given summary statistic and to compare individual loci to the genome-wide distribution under the assumption that loci subject to selection will be outliers [e.g. 78–84]. The rationale for this approach is that the demographic history of the population will shape patterns of variation genome-wide, so that the distribution of variation across loci will reflect the demographic history even if the actual history is unknown. Simulations under particular demographic models suggest that the false positive and false negative rate using this approach depends on the dominance of beneficial mutations and whether selection is acting on new mutations or standing variation [85]. A third approach is to use methods that account for population structure by estimating correlations among populations from the data directly [e.g. 8, 9]. These methods have the advantage of accounting for population history without requiring the specification of a (possibly incorrect) demographic model. A final approach is to sample populations over a known gradient of environmental factors and to look for clinal patterns of variation. This is a classic method that has been applied successfully to identify many of the best-studied examples of genes under selection [e.g., 30, 86].

Here we use a combination of several of these approaches. First, to account for the demographic history of the populations, we used LFMM [Latent Factor Mixed Model; 9], a computationally efficient program that implements a variant of Bayesian PCA in which residual population structure is introduced using unobserved (latent) factors. With this method, neutral population structure and covariance between environmental and genetic variation are simultaneously inferred. We initially explored settings for LFMM by running the program fifty times each for values of *K* (the number of latent factors) from two to five. Each run had a burn-in of 5,000 cycles of the Gibbs sampler algorithm and 10,000 iterations of the algorithm with latitude as the environmental factor. Results among runs with the same *K* were summarized using the R script provided in the LFMM manual. We then calculated the correlation among adjusted *p*-values for SNPs obtained for values of *K* ranging from two to five and evaluated the number of latent factors. Correlations were very high, with R^2^ values ranging from 0.89–0.99 and *K* = 2 was chosen based on a λ (genomic inflation factor) value close to one (λ = 0.81). We then ran LFMM 50 times with 50,000 burn-in cycles and 100,000 iterations of the Gibbs Sampler algorithm with *K* = 2, and z-scores were combined from the different runs using median values. Following the manual, *p*-values were adjusted to control for the false discovery rate (FDR). The distribution of *p*-values was examined and λ was modified to obtain a flatter distribution with a peak near zero (λ = 0.67; S9 Fig). A large pool of outlier SNPs were identified as those for which |z-score| ≥ 2 and each outlier SNP was annotated as having a |z-score| greater than or equal to two, three, or four. However, all SNPs with a |z-score| ≥3 had *q*-values < 0.05 after correction for multiple testing, thus a |z-score| ≥ 3 was chosen as the cutoff value in analyses of overlap with other methods. Candidate genes were identified as those containing outlier SNPs as annotated in GRCm38.75. In many cases, a single SNP had annotations for more than one gene, and all were included.

Second, we found that the five populations sampled in the eastern U.S. show no evidence of isolation by distance (S2 Fig). In other words, most polymorphisms in the genome do not vary in a clinal fashion. In contrast, many aspects of climate vary linearly with latitude (S1 Fig), suggesting that those polymorphisms that do vary clinally may be under environmentally mediated selection. Therefore, we compared individual loci to the genome-wide distribution of correlations between allele frequency and latitude for all variant sites using linear regression. We chose outliers according to two criteria. In the first case, we chose SNPs that were in the top 5% of the distribution of R^2^ and also in the top 5% of the distribution of the absolute value of the slope of the regression line, even when any one population was dropped from the analysis. Thus, these SNPs showed strong clinal patterns of variation with large frequency differences between the ends of the transect. These cut-offs resulted in outliers, when including all populations, with values of R^2^≥ 0.767 and values of |slope| ≥ 0.174, which translates into an allele frequency shift of ~44% or greater. These SNPs had a minimum R^2^≥ 0.743 and |slope| ≥ 0.167 when all populations were included or when any one population was excluded from the analysis (S3 Fig). Latitude was used as a proxy for climatic variation in all analyses due to its strong correlation with the first principal component summarizing climatic variables (Pearson’s r = -0.99, df = 3; *p*<0.0006; S18 Table). Candidate genes were identified as all genes for which outlier SNPs were annotated. Using the same regression approach, a second class of outliers was identified: all SNPs that were in the top 2.5% of the distribution of R^2^ of allele frequency with latitude, even when any one population was dropped from the analysis, regardless of slope. The rationale for this class of outliers is that covariance between allele frequency and environmental variables may be biologically meaningful, even in the absence of large changes in allele frequency. For example, such patterns are expected under a variety of conditions including selection on standing variation and on polygenic traits [31,33]. Such signals of selection might be missed by only focusing on genes showing major shifts in allele frequency. This criterion resulted in outliers with values of R^2^≥ 0.834 when all populations were included and a minimum R^2^≥ 0.830 including all populations or when any one population was dropped (S3 Fig). Candidate genes were identified as given above.

To address the effects of multiple testing, the minimum correlation coefficient and slope for each SNP were standardized to obtain z-scores (S10 Fig). As above, the minimum slope and correlation coefficient were determined by comparing values for each statistic when all populations were included and when any one population was excluded for a given SNP. The R package fdrtool [87] was then used to estimate *p*-values and *q*-values for each SNP using those z-scores (S11 Fig). Approximately 3% of all SNPs had *q*-values ≤ 0.01 for both correlation coefficient and slope. All of the SNPs identified as outliers with extreme correlation alone or with extreme correlation and slope had *q*-values ≤ 0.01 for the relevant statistic(s).

It should be noted that all methods that seek to identify genes under selection will be subject to false positives and false negatives. More stringent criteria will typically reduce the number of false positives at the cost of increasing the number of false negatives. Here, we have provided lists of genes that meet different criteria as a resource (S1 Data), but we have chosen to focus on those genes that contain outlier SNPs in LFMM and additionally show extreme correlation and allele frequency shifts with latitude. We then further narrow the field of candidates using the overlap between this set and those identified from whole-genome data, those harboring cis-eQTL, and those showing expression differences between mice from the ends of the transect (see below). This small set of genes are thus strong candidates for being targets of selection and are also associated with a known expression phenotype.

There was considerable overlap between the candidate genes identified using LFMM and those identified with linear regression. To test whether the overlap was more than expected by chance, we randomly sampled (without replacement) the same number of genes from each candidate list from a list of all of the genes sampled in our exomic data. We then calculated the overlap between each pair of methods and all three methods. We repeated this 10,000 times. In all cases, the observed overlap was more extreme than any overlap from the random samples.

### Estimating the distance over which signals extend in the exome

Estimating the distance over which signals of environmental adaptation extend is complicated by the nature of exome data that are necessarily limited to regions in or near genes. Moreover, while genomic data were generated, this was done at low coverage preventing the use of methods for estimating linkage disequilibrium that rely on calling individual genotypes. In order to approach this question, we used the exome data and identified the SNP for which the LFMM |z-score| was the highest in each candidate gene. When multiple genes were included as candidates as the result of a SNP or group of SNPs that were annotated to multiple genes, only one gene was included in the analysis. We then identified the maximum |z-score| for windows of 2kb starting 50 bp upstream or downstream and ending at 36kb upstream or downstream. If there were no data in a window, we continued to the next window. For each gene, we recorded the first window upstream and downstream in which there were data and the first in which the maximum |z-score| dropped below 3. We found that signals of selection do not generally extend over long genomic distances. The signal of selection extends less than 25kb upstream and downstream in > 70% of the genes identified by all three cut-offs (Fig 2A; S11 Table). We then repeated the analysis with a maximum |z-score| of 2. In general, signals largely dropped off within 22 kb (S11 Table).

### Identifying the potential effects of candidate SNPs in the exome

The potential functional consequence of each SNP was determined using Ensembl’s variant effect predictor [88]. SNPs often had more than one potential effect and all were included in annotation. To determine the distribution of functional consequences among all SNPs and among all candidate SNPs, a primary functional consequence was designated for each SNP. The primary consequence was determined based on the minimum rank of all the annotations for a SNP using the following scheme:

1. Missense, stop lost, or stop gained
2. 3’ or 5’ UTR
3. Synonymous
4. Non-coding exon variant, Non-coding transcript variant, and/or any other coding variant
5. Intronic or splice site variants
6. Any remaining non-coding variants
7. Upstream or downstream variants

The distribution of candidate SNPs among different potential effect categories was similar for regression and LFMM (S12 Table).

### Identifying candidate regions contributing to environmental adaptation in the whole genome

We used linear regression of allele frequency and latitude to calculate R^2^ and |slope| for each SNP that passed all filters. To identify regions of interest, we then used three different window analyses: 1000 bp windows with a step size of 500 bp, 1500 bp windows with a step size of 750 bp, and 2500 bp windows with a step size of 2500 bp. Windows were only included in analyses when they had at least three SNPs. The cut-off values for R^2^ and |slope| were determined from the 95% percentile of the average values for those statistics calculated when all populations were included and when any one population was excluded. Outlier windows were identified as those with average values of R^2^ and |slope| that met or exceeded the cut-off values when all populations were included and when any one population was excluded. For example, for the 2500 bp window analysis, there were >715,000 windows for which there were sufficient data. Less than 0.3% of those windows, ~2,100, were identified as outliers with $\left|\bar{slope}\right|$ ≥ 0.153 and $\bar{R^{2}}$ ≥ 0.566 (S12 Fig). To determine whether these candidate regions fell in or near genes, we used custom PERL scripts to identify when any candidate window fell within ± 5kb of a feature in GRCm38.75 (S14 Table) or when any window overlapped putative promoters (± 500 bp) from the Mouse ENCODE project [36]. Over 1,500 genes were identified in the candidate regions from the three different window analyses combined (S1 Data).

Of the 177 genes that were identified in all exome approaches, 171 are autosomal, and 127 of those were identified in the genome analysis. To test whether the overlap was more than expected by chance, we randomly sampled (without replacement) 171 genes from the autosomal genes sampled in our exome data and calculated the overlap with genes identified in the genome. We repeated this 10,000 times. In all cases, the observed overlap was more extreme than any overlap from the random samples (permutation test, *p* < 0.0001). Repeating with replacement did not change the results.

### Patterns of gene expression in lab-reared mice

We compared gene expression in mice derived from wild populations at the northern (New York) and southern (Florida) ends of the transect. First, we focused on three tissues in N_1_ males: the liver, the hypothalamus and the dorsal, hind limb fat pad. Four unrelated males from each location were included. The mice ranged in age from 99–143 days. All were unmated and housed singly in a common laboratory environment with the same diet. Second, we focused on liver tissue in N_2_ males. While differences among populations in gene expression in the N_1_ generation cannot be attributed to environmental differences directly, expression differences could be due, in some part, to conditions experienced by wild caught mothers. Evaluating gene expression in the N_2_ animals can address the potential impact of maternal effects. Four unrelated male N_2_ mice from each location were included and they ranged in age from 149–210 days. All animals were sacrificed at the same time of day, and tissue was collected and either flash frozen in liquid nitrogen or submerged in RNAlater prior to storage at -80°C.

RNA was extracted from liver tissue using the Qiagen RNeasy Plus kit and from adipose tissue and the hypothalamus using the Quiagen RNeasy Lipid Tissue Kit with a genomic DNA digestion. RNA quality was verified using a Bioanalyzer (Agilent) or a Fragment Analyzer (Advanced Analytic Technologies). Libraries were prepared following ribo-depletion at the University of California, Davis DNA Technologies and Expression Analysis Cores Genome Center. All libraries were pooled and run on two lanes of the HiSeq3000 (100 bp paired-end) resulting in >2.5 GB of raw data per sample. Reads were trimmed using Trimmomatic [89]. The resulting reads were mapped to the mouse genome (GRCm38) using TopHat v2.0.13 [90,91]. Reads that mapped to multiple locations were removed and HTseq [92] was used to summarize count data for each feature using the .gtf file associated with GRCm38.

DESeq 2 [93] was used to identify genes with significant differences in expression between the descendants of wild-caught mice from New York and Florida. First, we used PCA to explore differences in patterns of gene expression after transforming the data using the rlog function in DESeq2 to account for the positive relationship between mean values and variance in gene expression data (S5 Fig). PCA clearly distinguished the two populations in all tissues, including the N_2_ liver. The persistence of differences into the second generation in the lab suggests that observed differences in gene expression are not likely to be due to maternal effects. PCA was also used to identify outliers including one individual in the N_2_ liver analysis and two individuals in the N_1_ fat analysis (S5 and S6 Figs). In the first case, a single individual was outside of the range of all individuals from both populations on the first principle component axis, which explained 30% of the variance in gene expression (S5C and S6A Figs). That individual was excluded from all further analysis of differential gene expression. In the second case, N_1_ fat, we found that a single individual from each population clustered with the opposite population (S5D Fig). Because we analyzed several tissues from the same individuals in the N_1_, we were able to use sequence variants to confirm that individuals were labeled correctly. Interestingly, we found that the two individuals who appeared “mismatched” in the fat analysis, were also outliers in phenotype (S13 Fig); it was the leanest mouse from New York (as measured by mass divided by length) that clustered with Florida, and the fattest mouse from Florida that clustered with New York. These two individuals were excluded from further analysis of differential expression (S6B Fig), but underscore the biological connection between gene expression and phenotype. Finally, gene-wise tests of differential expression were implemented in DESeq2 with the default correction for multiple testing. When identifying genes with evidence of selection and differential expression, a permissive cut-off of *p*_*adj*_<0.10 was used. While many genes were differentially expressed in fat, we found a modest number of genes with evidence of differential expression in the other tissues (S15 Table).

### Identifying cis-eQTL using expression data from lab-reared mice

Allelic imbalance, a difference in expression between two alleles at a locus, can be used to identify *cis-* regulatory variation in gene expression [37]. While *trans*- acting variants affect the expression of both alleles in a cell, *cis-* regulatory variants affect expression in an allele-specific manner. As a consequence, differences in the expression of two alleles at a heterozygous site within an individual can be used to infer *cis*- regulatory variation. We used the RNAseq data to identify *cis*- regulatory variation in lab-born progeny of individuals from Florida and New York. Variants were called with samtools mpileup version 1.3.1 [94] and bcftools version 1.3.1, requiring a minimum mapping quality score of 20 and a Phred-scaled quality (Q) score of 30. Mapping bias towards the reference allele may reduce the accuracy of allele-specific expression measurements [95]. To mitigate the effects of reference mapping bias, these genotype calls were used to create personal reference genomes for each sample [96]. Heterozygous sites were masked by inserting “Ns” in the mouse genome using bedtools [97]. While only heterozygous sites were used in the downstream allele-specific expression analysis, indels were also masked because these sites can cause biased allele-specific assignment [98]. Pre-processed reads were then re-mapped to personal reference genomes with TopHat v2.0.13 [90, 91]. After re-mapping, only uniquely mapped reads that overlapped exonic heterozygous sites were retained for further analysis. Sites present in more than one gene were removed from the analysis. Downsampling of allele-specific reads was used to equalize power [98]. Sites where more than 20 reads mapped to both the reference and alternative allele were tested for allelic imbalance [99]. Binomial exact tests were used to identify significant differences in relative allelic expression. Sites within 350 bp and in the same gene were then grouped. The lowest *p*-value in each group was corrected to a 10% false discovery rate (FDR). We found many genes with evidence for *cis*-eQTL (S15 Table).

### Functional information

A wealth of data is available on gene function in mice including phenotypic evaluation of mice with gene knockouts or mutations, associations with human disease, gene ontologies, QTL studies, and pathway maps. To explore the potential functional significance of candidates identified in our analyses, we used MouseMine to query for associated phenotypes, human diseases, gene ontologies (GO), and overlapping QTL [34, 35]. We also used KEGG to identify all pathways in which candidate genes were included [100, 101]. Phenotype summary information (Table 1, S16 Table) was collated by searching mammalian phenotype terms, GO terms, KEGG pathways, and overlapping QTL for each gene for terms related to the category of interest, as follows:

1. Circadian Rhythm: known clock genes [see 102, 103], GO terms, phenotype terms, or QTL with clock or circadian.
2. Fat: GO terms or phenotype terms with fat or adipose, hand-curated; QTL with fat, adipose, obese, obesity, or body mass index, hand-curated.
3. Body Size: Phenotype terms with body, hand-curated; QTL with body, weight and weeks, or growth, hand-curated.
4. Immunity: Phenotype or GO term with immun*; QTL or phenotype terms with resistance or suscept*, hand-curated.
5. Blood Chemistry/Diabetes: KEGG pathway adipocytokine signaling, ampk signaling (relating to leptin, adiponectin); QTL, phenotype terms, or GO terms with diab*, nidd, gluc*, leptin, cholesterol, adiponectin; phenotype or GO terms with clot or coagulation.
6. Nesting: Phenotype term nesting behavior.

Summary categories presented were chosen due to potential links to phenotypes that vary in this study (e.g. body size, blood chemistry) or to traits that potentially could vary over large geographic distances (e.g. immunity, circadian rhythm).

### Overlap with results of analyses in human populations

We collated a list of genes associated with environmental adaptation in humans [60–62]. Forty-three of those genes had one-to-one orthologs in mice. Of those, 18 were identified as candidates using LFMM with a cut-off of |z-score| ≥ 2 (S17 Table). To determine if the overlap between LFMM outliers in our study and the genes identified in humans was more than expected by chance, we randomly sampled (without replacement) the same number of genes as were identified using that same cut-off from among all genes sampled in our exomic data. For all of these analyses, only genes with one-to-one orthologs in humans were included. We then calculated the overlap between the candidate genes from human studies and the random selection of genes. We repeated this 10,000 times. Outcomes equal to or more extreme than the observed overlap of 18 genes occurred in 2.56% of the samples.

### Accession numbers

Sequence data can be accessed via the NCBI SRA under BioProject IDs: PRJNA397150 –exome, PRJNA397406 –genome, PRJNA412620 –RNA-Seq.

## Supporting information

S1 Table Correlation between latitude and measures of body size for wild-caught mice from the transect. (DOCX)

S2 Table Results of analysis of body mass, body length, and BMI across generations including wild-caught, N_1_, and N_2_ individuals from NY and FL. (DOCX)

S3 Table Average, standard deviation, and sample size by population (FL,NY) and generation for each phenotype analysis. (DOCX)

S4 Table Results of analysis of variance in body mass, body length, and BMI for N_2_ (n = 147) mice from NY and FL. (DOCX)

S5 Table Results of a linear mixed effects model analysis of aspects of blood chemistry in N_2_ mice from NY and FL (n = 40). (DOCX)

S6 Table Results of analysis of food intake in N_2_ mice from NY and FL (n = 64). (DOCX)

S7 Table Results of analysis of nest-building in N_2_ mice from NY and FL (n = 64). (DOCX)

S8 Table Results of analysis of wheel-running activity in N_2_ mice from from NY and FL (n = 72). (DOCX)

S9 Table Yields of data obtained via HiSeq2000 sequencing of genomic libraries enriched for exomic regions via Nimblegen SeqCap EZ capture array pre- and post- processing. (DOCX)

S10 Table Pairwise differentiation (*F*_*st*_) and geographic distance (km) among surveyed populations. (DOCX)

S11 Table The number of candidate genes identified in all three methods in the exome that show evidence of a drop-off of the signal of selection within a given window upstream and downstream of the primary candidate SNP. (DOCX)

S12 Table The primary annotation for candidate SNPs identified via different methods and in the full dataset. (DOCX)

S13 Table Yields of data obtained via HiSeq2000 sequencing of genomic libraries. (DOCX)

S14 Table Number of candidate windows identified in the genome that fall in or near genes and/or near putative promoters. (DOCX)

S15 Table The number of genes with evidence of differential expression or allele specific expression in lab raised mice derived from wild populations in New York and Florida. (DOCX)

S16 Table Genes identified as candidates in the genome analyses that also show evidence of differential expression and allele specific expression in the same tissue. (DOCX)

S17 Table Overlap between genes identified as candidates for environmental adaptation in humans and in our study. (DOCX)

S18 Table Loading matrix and values for the first four principal components summarizing climate variables. (DOCX)

S19 Table Estimates of nucleotide diversity for the surveyed populations. (DOCX)

S1 Fig Climate variables versus latitude for locations included in the transect. (DOCX)

S2 Fig There is no evidence of a significant association between genetic distance and geographic distance (A) Geographic distance vs. pairwise F_st_ (B) genetic PCA.
(DOCX)

S3 Fig The distribution of (A) R^2^ and (B) |slope| for the linear relationship between allele frequency and latitude for exomic SNPs.
(DOCX)

S4 Fig The correlation between allele frequency estimates from the exomic and genomic data (A) given the entire sample of 50 individuals (B) given individual populations.
(DOCX)

S5 Fig The first two principal components of variation in gene expression data from four tissues in male mice (A) hypothalamus N_1_ (B) liver N_1_ (C) liver N_2_ (D) Fat N_1_.
(DOCX)

S6 Fig The first two principal components of variation in gene expression data from the **(A)** liver of N_2_ male mice and (B) fat of N_1_ male mice after removal of outliers.
(DOCX)

S7 Fig The distribution of *F*_*st*_ estimates for all genes and for different sets of candidate genes in the exome. (DOCX)

S8 Fig Candidate gene *Mc3r* (A) Exome data show that allele frequencies for SNPs in *Mc3r* are highly correlated with latitude. (B) QTL and (C) knock-out mouse strains show that there are functional links between *Mc3r* and phenotypes that differ in our study among mice from different latitudes.
(DOCX)

S9 Fig The distribution of adjusted *p*-values for LFMM given *K* = 2 after modifying the genome inflation factor (λ). (DOCX)

S10 Fig Distribution of (A) the minimum correlation coefficient (B) the standardized minimum correlation coefficient (C) the minimum slope and (D) the standardized minimum slope for the linear relationship between allele frequencies of SNPs from the exome and latitude.
(DOCX)

S11 Fig The distribution of (A) *p*-values and (B) *q*-values of the z-scores of the minimum correlation coefficient for SNPs in the exome and the distribution of (C) *p*-values and (D) *q*-values of the z-scores of the minimum slope for SNPs in the exome.
(DOCX)

S12 Fig The distribution of (A) average R^2^ values and (B) average |slope| for 2500 bp windows in the genome.
(DOCX)

S13 Fig Body weight divided by body length for male mice from FL (red) and NY (blue) included in the expression study. (DOCX)

S1 Data Excel file containing supporting data on collections, climate, phenotypes, and expression as well as a summary table for data on all candidate genes. (XLSX)
