# Supplementary material for: The genomic basis of environmental adaptation in house mice
Source: PLoS Genet. 2018 Sep 24;14(9):e1007672. doi: 10.1371/journal.pgen.1007672 (PMC6171964; doi:10.1371/journal.pgen.1007672)
Supplement: S1 Table — (DOCX) [file pgen.1007672.s001.docx]

Supplementary Table 1. Correlation between latitude and measures of body size for wild-caught mice from the transect. Pregnant and/or lactating mice and one juvenile were excluded.

|  |  | Body Mass (g) | | Body Length (mm) | | Body Mass (g)/  Body Length (mm) | | BMI (kg/m^2^) | |
| --- | --- | --- | --- | --- | --- | --- | --- | --- | --- |
| Sex | n | Pearson’s *r* | *P* | Pearson’s *r* | *P* | Pearson’s *r* | *P* | Pearson’s *r* | *P* |
| All | 45 | 0.426 | 0.004^**^ | 0.306 | 0.041^*^ | 0.422 | 0.004^**^ | 0.303 | 0.043^*^ |
| Female | 18 | 0.492 | 0.038^*^ | 0.338 | 0.171 | 0.495 | 0.037^*^ | 0.346 | 0.160 |
| Male | 27 | 0.379 | 0.051 | 0.280 | 0.158 | 0.376 | 0.054 | 0.286 | 0.148 |

^*^P<0.05, ^**^P<0.01
