# Supplementary material for: The genomic basis of environmental adaptation in house mice
Source: PLoS Genet. 2018 Sep 24;14(9):e1007672. doi: 10.1371/journal.pgen.1007672 (PMC6171964; doi:10.1371/journal.pgen.1007672)
Supplement: S2 Table — (DOCX) [file pgen.1007672.s002.docx]

Supplementary Table 2. Results of analysis of body mass, body length, and BMI across generations including wild-caught, N_1_, and N_2_ individuals from NY and FL (n=351). The GLM was of the form: Response Variable~Generation*Population*Sex. Significant interaction terms are reported.

| Response Variable | Predictor | Df | Sums of Squares | Mean Square | F | *P* value |
| --- | --- | --- | --- | --- | --- | --- |
| Body Mass | Population | 1 | 1130.74 | 1130.74 | 74.49 | 2.43 x 10^-16****^ |
|  | Sex | 1 | 982.89 | 982.89 | 64.75 | 1.46 x 10^-14****^ |
|  | Generation | 2 | 1.76 | 0.88 | 0.06 | 0.944 |
|  | Population:Generation | 2 | 201.63 | 100.81 | 6.64 | 0.002^**^ |
|  | Residuals | 338 | 5130.60 | 15.18 |  |  |
| Body Length | Population | 1 | 1997.32 | 1997.32 | 27.01 | 3.51 x 10^-7****^ |
|  | Sex | 1 | 98.77 | 98.77 | 1.34 | 0.249 |
|  | Generation | 2 | 32.26 | 16.13 | 0.22 | 0.804 |
|  | Population:Generation | 2 | 2041.74 | 1020.87 | 13.81 | 1.73 x 10^-6****^ |
|  | Residuals | 338 | 24995.00 | 73.95 |  |  |
| BMI | Population | 1 | 2.30 | 2.30 | 11.87 | 6.44 x 10^-4***^ |
|  | Sex | 1 | 11.05 | 11.05 | 56.90 | 4.33 x 10^-13****^ |
|  | Generation | 2 | 0.24 | 0.12 | 0.62 | 0.540 |
|  | Residuals | 338 | 65.65 | 0.19 |  |  |
| Body Mass/Length | Population | 1 | 0.07 | 0.07 | 53.98 | 1.53 x 10^-12****^ |
|  | Sex | 1 | 0.10 | 0.10 | 83.11 | <1.00 x 10^-15****^ |
|  | Generation | 2 | 4.07 x 10^-4^ | 2.04 x 10^-4^ | 0.17 | 0.85 |
|  | Residuals | 338 | 0.41 | 1.21 x 10^-3^ |  |  |

^*^*P*<0.05, ^**^*P* <0.01, ^***^*P* <0.001, ^****^*P* <0.0001
