# Supplementary material for: The genomic basis of environmental adaptation in house mice
Source: PLoS Genet. 2018 Sep 24;14(9):e1007672. doi: 10.1371/journal.pgen.1007672 (PMC6171964; doi:10.1371/journal.pgen.1007672)
Supplement: S3 Table — (DOCX) [file pgen.1007672.s003.docx]

Supplementary Table 3. Average (AVG), standard deviation (SD), and sample size (n) by population (FL=Florida, NY=New York) and generation for each phenotype analysis.

|  |  |  | FL | | | NY | | |
| --- | --- | --- | --- | --- | --- | --- | --- | --- |
|  | Generation(s) | units | AVG | SD | n | AVG | SD | n |
| Body Mass | N_0_ | gm | 18.53 | 3.29 | 49 | 21.33 | 4.72 | 21 |
| Body Mass | N_1_ | gm | 17.15 | 3.07 | 56 | 22.70 | 4.90 | 77 |
| Body Mass | N_2_ | gm | 18.80 | 4.09 | 84 | 20.98 | 4.94 | 63 |
| Body Length | N_0_ | mm | 86.19 | 6.68 | 49 | 93.21 | 6.25 | 21 |
| Body Length | N_1_ | mm | 83.73 | 10.31 | 56 | 93.74 | 9.96 | 77 |
| Body Length | N_2_ | mm | 89.74 | 8.79 | 84 | 89.05 | 6.45 | 63 |
| Body Mass/Body Length | N_0_ | gm/mm | 0.21 | 0.03 | 49 | 0.23 | 0.015 | 21 |
| Body Mass/Body Length | N_1_ | gm/mm | 0.21 | 0.03 | 56 | 0.24 | 0.04 | 77 |
| Body Mass/Body Length | N_2_ | gm/mm | 0.21 | 0.04 | 84 | 0.23 | 0.04 | 63 |
| BMI | N_0_ | kg/m^2^ | 2.49 | 0.33 | 49 | 2.48 | 0.66 | 21 |
| BMI | N_1_ | kg/m^2^ | 2.49 | 0.53 | 56 | 2.59 | 0.45 | 77 |
| BMI | N_2_ | kg/m^2^ | 2.35 | 0.49 | 84 | 2.63 | 0.45 | 63 |
| Adiponectin | N_2_ | µg/mL | 24.46 | 10.86 | 20 | 30.28 | 11.47 | 20 |
| Cholesterol, Total | N_2_ | mg/dL | 122.35 | 39.35 | 20 | 124.38 | 25.87 | 20 |
| Cholesterol, HDL | N_2_ | mg/dL | 37.28 | 12.45 | 20 | 44.85 | 21.73 | 20 |
| Free Fatty Acids | N_2_ | mEq/dL | 1.06 | 0.08 | 20 | 1.05 | 0.15 | 20 |
| Glucose | N_2_ | mg/dL | 139.55 | 39.58 | 20 | 119.79 | 54.07 | 20 |
| Insulin | N_2_ | pg/mL | 221.86 | 140.57 | 20 | 1090.55 | 2312.47 | 20 |
| Leptin | N_2_ | pg/mL | 1039.65 | 955.07 | 20 | 515.70 | 432.16 | 20 |
| Triglycerides | N_2_ | mg/dL | 44.61 | 37.08 | 20 | 25.76 | 14.50 | 20 |
| Food intake | N_2_ | gm | 4.32 | 3.23 | 39 | 5.39 | 4.73 | 25 |
| Nest Weight | N_2_ | gm | 5.76 | 5.51 | 39 | 12.19 | 11.60 | 25 |
| Wheel Running Score | N_2_ | log(km+1) ^*^ | 0.71 | 0.47 | 34 | 1.02 | 0.51 | 38 |

^*^Distance was corrected for slight differences in run time among animals
