# Supplementary material for: The genomic basis of environmental adaptation in house mice
Source: PLoS Genet. 2018 Sep 24;14(9):e1007672. doi: 10.1371/journal.pgen.1007672 (PMC6171964; doi:10.1371/journal.pgen.1007672)
Supplement: S4 Table — (DOCX) [file pgen.1007672.s004.docx]

Supplementary Table 4. Results of analysis of variance in body mass, body length, and BMI for N_2_ (n=147) mice from NY and FL. The GLM was of the form: Response Variable ~ Population * Sex * Age. Significant interaction terms are reported.

| Response Variable | Predictor | Df | Sums of Squares | Mean Square | F | *P* |
| --- | --- | --- | --- | --- | --- | --- |
| Body Mass | Population | 1 | 170.69 | 170.69 | 11.49 | 9.13 x 10^-4***^ |
|  | Sex | 1 | 443.96 | 443.96 | 29.88 | 2.07 x 10^-7****^ |
|  | Age | 1 | 283.20 | 283.20 | 19.06 | 2.46 x 10^-5****^ |
|  | Population:Age | 1 | 78.47 | 78.47 | 5.28 | 0.023^*^ |
|  | Residuals | 139 | 2065.60 | 14.86 |  |  |
| Body Length | Population | 1 | 17.46 | 17.46 | 0.39 | 0.533 |
|  | Sex | 1 | 94.96 | 94.96 | 2.12 | 0.148 |
|  | Age | 1 | 2484.19 | 2484.19 | 55.50 | 9.00 x 10^-12****^ |
|  | Residuals | 139 | 6221.8 | 44.76 |  |  |
| BMI | Population | 1 | 2.80 | 2.80 | 14.68 | 1.92 x 10^-4***^ |
|  | Sex | 1 | 4.24 | 4.24 | 22.24 | 5.78 x 10^-6****^ |
|  | Age | 1 | 0.13 | 0.13 | 0.66 | 0.418 |
|  | Population:Age | 1 | 1.22 | 1.22 | 6.41 | 0.012^*^ |
|  | Residuals | 139 | 26.48 | 0.19 |  |  |
| Body Mass/Length | Population | 1 | 0.02 | 0.02 | 16.59 | 7.77 x 10^-5****^ |
|  | Sex | 1 | 0.04 | 0.04 | 31.63 | 9.89 x 10^-8****^ |
|  | Age | 1 | 5.95 x 10^-3^ | 5.95 x 10^-3^ | 4.25 | 0.041^*^ |
|  | Population:Age | 1 | 9.97 x 10^-3^ | 9.97 x 10^-3^ | 7.12 | 0.009^**^ |
|  | Residuals | 139 | 0.20 | 1.44 x 10^-3^ |  |  |

^*^*P*<0.05, ^**^*P* <0.01, ^***^*P* <0.001, ^****^*P* <0.0001
