# Supplementary material for: The genomic basis of environmental adaptation in house mice
Source: PLoS Genet. 2018 Sep 24;14(9):e1007672. doi: 10.1371/journal.pgen.1007672 (PMC6171964; doi:10.1371/journal.pgen.1007672)
Supplement: S5 Table — (DOCX) [file pgen.1007672.s005.docx]

Supplementary Table 5. Results of a linear mixed effects model analysis of aspects of blood chemistry in N_2_ mice from NY and FL (n=40). All models were of the form: log(trait)~Population+Sex+log(mass)+log(length)+(1|Family).

|  | Predictor | Df | χ^2^ | *P* |
| --- | --- | --- | --- | --- |
| Adiponectin | Population | 1 | 3.96 | 0.046^*^ |
|  | Sex | 1 | 39.30 | <0.001^***^ |
|  | log(mass) | 1 | 0.19 | 0.664 |
|  | log(length) | 1 | 2.34 | 0.126 |
| Cholesterol, Total | Population | 1 | 0.10 | 0.752 |
|  | Sex | 1 | 0.08 | 0.774 |
|  | log(mass) | 1 | 1.75 | 0.185 |
|  | log(length) | 1 | 0.00 | 1.000 |
| Cholesterol, HDL | Population | 1 | 0.88 | 0.348 |
|  | Sex | 1 | 0.17 | 0.677 |
|  | log(mass) | 1 | 0.03 | 0.872 |
|  | log(length) | 1 | 3.80 | 0.051 |
| Free Fatty Acids | Population | 1 | 0.00 | 0.955 |
|  | Sex | 1 | 1.25 | 0.263 |
|  | log(mass) | 1 | 2.68 | 0.101 |
|  | log(length) | 1 | 1.67 | 0.196 |
| Glucose | Population | 1 | 4.82 | 0.028^*^ |
|  | Sex | 1 | 0.82 | 0.364 |
|  | log(mass) | 1 | 10.46 | 0.001^***^ |
|  | log(length) | 1 | 5.00 | 0.025^*^ |
| Insulin | Population | 1 | 0.18 | 0.670 |
|  | Sex | 1 | 1.57 | 0.211 |
|  | log(mass) | 1 | 0.17 | 0.679 |
|  | log(length) | 1 | 0.14 | 0.709 |
| Leptin | Population | 1 | 6.59 | 0.010^**^ |
|  | Sex | 1 | 6.38 | 0.012^*^ |
|  | log(mass) | 1 | 13.58 | <0.001^**^ |
|  | log(length) | 1 | 2.44 | 0.118 |
| Triglycerides | Population | 1 | 5.10 | 0.024^*^ |
|  | Sex | 1 | 0.50 | 0.481 |
|  | log(mass) | 1 | 2.41 | 0.120 |
|  | log(length) | 1 | 0.11 | 0.740 |

^*^*P* <0.05, ^**^*P* <0.01, ^***^*P* <0.001
