# Supplementary material for: The genomic basis of environmental adaptation in house mice
Source: PLoS Genet. 2018 Sep 24;14(9):e1007672. doi: 10.1371/journal.pgen.1007672 (PMC6171964; doi:10.1371/journal.pgen.1007672)
Supplement: S6 Table — (DOCX) [file pgen.1007672.s006.docx]

Supplementary Table 6. Results of analysis of food intake in N_2_ mice from NY and FL (n=64). The GLM was of the form: Food Intake ~ Population + Sex + Body Mass.

| Predictor | Df | Sums of Squares | Mean Square | F | *P* |
| --- | --- | --- | --- | --- | --- |
| Population | 1 | 17.55 | 17.55 | 1.25 | 0.267 |
| Sex | 1 | 1.28 | 1.28 | 0.19 | 0.764 |
| Body Mass  Residuals | 1  60 | 92.12  839.59 | 92.12  13.99 | 6.58 | 0.013^*^ |

^*^*P* <0.05
