# Supplementary material for: The genomic basis of environmental adaptation in house mice
Source: PLoS Genet. 2018 Sep 24;14(9):e1007672. doi: 10.1371/journal.pgen.1007672 (PMC6171964; doi:10.1371/journal.pgen.1007672)
Supplement: S7 Table — (DOCX) [file pgen.1007672.s007.docx]

Supplementary Table 7. Results of analysis of nest-building in N_2_ mice from NY and FL (n=64). The GLM was of the form: Nest Weight ~ Population + Sex + Body Mass.

| Predictor | Df | Sums of Squares | Mean Square | F | *P* |
| --- | --- | --- | --- | --- | --- |
| Population | 1 | 628.83 | 628.83 | 9.93 | 0.003^**^ |
| Sex | 1 | 6.20 | 6.20 | 0.10 | 0.756 |
| Body Mass  Residuals | 1  60 | 574.22  3800.50 | 574.22  63.34 | 9.07 | 0.004^**^ |

^*^*P* <0.05, ^**^*P* <0.01
