# Supplementary material for: The genomic basis of environmental adaptation in house mice
Source: PLoS Genet. 2018 Sep 24;14(9):e1007672. doi: 10.1371/journal.pgen.1007672 (PMC6171964; doi:10.1371/journal.pgen.1007672)
Supplement: S8 Table — (DOCX) [file pgen.1007672.s008.docx]

Supplementary Table 8. Results of analysis of wheel-running activity in N_2_ mice from from NY and FL (n=72). The GLM was of the form: Wheel Score ~Population + Sex.

| Predictor | Df | Sums of Squares | Mean Square | F | *P* |
| --- | --- | --- | --- | --- | --- |
| Population | 1 | 1.74 | 1.74 | 7.22 | 0.009^**^ |
| Sex | 1 | 0.21 | 0.21 | 0.86 | 0.358 |
| Residuals | 69 | 16.60 | 0.24 |  |  |

^*^*P* <0.05, ^**^*P* <0.01
