# Supplementary material for: The genomic basis of environmental adaptation in house mice
Source: PLoS Genet. 2018 Sep 24;14(9):e1007672. doi: 10.1371/journal.pgen.1007672 (PMC6171964; doi:10.1371/journal.pgen.1007672)
Supplement: S9 Table — (DOCX) [file pgen.1007672.s009.docx]

Supplementary Table 9. Yields of data obtained via HiSeq2000 sequencing of genomic libraries enriched for exomic regions via Nimblegen SeqCap EZ capture array pre- and post- processing.

| Individual | Raw Data (Mb) | Cleaned Data (Mb) | Data Mapped (Mb) | Specificity (%) | Sensitivity (%) | Average Coverage(X) | Sites 5X (%) | Sites 10X (%) | Sites 20X (%) |
| --- | --- | --- | --- | --- | --- | --- | --- | --- | --- |
| MPR_108 | 1400.73 | 1171.39 | 737.43 | 62.95 | 91.88 | 10.39 | 72.97 | 44.3 | 12.86 |
| MPR_110 | 1920.1 | 1605.38 | 1015.03 | 63.23 | 92.58 | 14.28 | 79.57 | 58.12 | 24.91 |
| MPR_112 | 1635.25 | 1387.2 | 872.72 | 62.91 | 92.36 | 12.29 | 76.48 | 51.58 | 19.04 |
| MPR_113 | 1494.51 | 1280.94 | 795.31 | 62.09 | 92.18 | 11.23 | 74.65 | 47.96 | 15.64 |
| MPR_114 | 2504.03 | 2041.82 | 1345.66 | 65.9 | 93.36 | 18.78 | 84.87 | 69.22 | 37.84 |
| MPR_115 | 1960.3 | 1635.2 | 1026.34 | 62.77 | 92.67 | 14.5 | 79.88 | 58.5 | 25.33 |
| MPR_116 | 1663.93 | 1408.69 | 875.08 | 62.12 | 92.29 | 12.35 | 77.05 | 52.38 | 19.09 |
| MPR_118 | 1816.25 | 1532.14 | 963.22 | 62.87 | 92.79 | 13.59 | 78.6 | 56.05 | 22.84 |
| MPR_120 | 1554.36 | 1305.3 | 797.88 | 61.13 | 92.27 | 11.31 | 74.96 | 48.37 | 15.8 |
| MPR_121 | 3195.67 | 2640.19 | 1705.77 | 64.61 | 93.55 | 24.28 | 82.68 | 68.55 | 45.65 |
| MPR_123 | 1502.92 | 1265.59 | 771.54 | 60.96 | 92.02 | 10.98 | 73.78 | 46.91 | 14.9 |
| MPR_124 | 1635.61 | 1382.54 | 843.46 | 61.01 | 92.51 | 12.06 | 75.59 | 49.89 | 17.96 |
| MPR_125 | 1707.87 | 1445.25 | 891.43 | 61.68 | 92.7 | 12.65 | 77.65 | 53.51 | 19.73 |
| MPR_126 | 2157.46 | 1833.61 | 1163.26 | 63.44 | 91.89 | 16.54 | 76.76 | 59.52 | 31.76 |
| MPR_128 | 1554.36 | 1314 | 819.45 | 62.36 | 92.08 | 11.61 | 75.13 | 49.23 | 16.66 |
| MPR_129 | 1776.17 | 1498.7 | 936.7 | 62.5 | 92.75 | 13.26 | 78.25 | 55.01 | 21.55 |
| MPR_130 | 2012.5 | 1673.83 | 1041.56 | 62.23 | 92.87 | 14.73 | 80.62 | 60 | 26.45 |
| MPR_131 | 2272.31 | 1862.59 | 1180.63 | 63.39 | 89.75 | 16.94 | 69.45 | 52.69 | 30.86 |
| MPR_132 | 2782.95 | 2253.41 | 1486.75 | 65.98 | 83.5 | 21.3 | 60.26 | 49.16 | 35.23 |
| MPR_133 | 2087.59 | 1636.79 | 986.77 | 60.29 | 93.34 | 14.14 | 80.26 | 57.75 | 23.97 |
| MPR_134 | 1972.53 | 1639.54 | 1037.52 | 63.28 | 92.96 | 14.58 | 81.13 | 59.99 | 25.89 |
| MPR_135 | 3050.1 | 2506.31 | 1624.68 | 64.82 | 92.74 | 22.92 | 83.33 | 70.95 | 46.03 |
| MPR_137 | 1801.08 | 1486.42 | 938.34 | 63.13 | 92.51 | 13.24 | 78.23 | 54.93 | 21.49 |
| MPR_138 | 1948.46 | 1573.74 | 963.85 | 61.25 | 90.88 | 13.92 | 68.25 | 47.62 | 23.8 |
| MPR_140 | 2654.44 | 2189.54 | 1378.39 | 62.95 | 93.44 | 19.46 | 84.74 | 70.19 | 40.05 |
| MPR_141 | 1908.4 | 1598.32 | 1014.88 | 63.5 | 92.77 | 14.27 | 80.03 | 58.44 | 25.03 |
| MPR_142 | 1647.63 | 1376.39 | 875.55 | 63.61 | 92.43 | 12.32 | 77.23 | 52.17 | 18.62 |
| MPR_143 | 2611.85 | 2117.28 | 1347.82 | 63.66 | 93.04 | 19.3 | 79.07 | 60.19 | 33.59 |

Supplementary Table 9, continued. Yields of data obtained via HiSeq2000 sequencing of genomic libraries enriched for exomic regions via Nimblegen SeqCap EZ capture array pre- and post- processing.

| Individual | Raw Data (Mb) | Cleaned Data (Mb) | Data Mapped (Mb) | Specificity (%) | Sensitivity (%) | Average Coverage (X) | Sites 5X (%) | Sites 10X (%) | Sites 20X (%) |
| --- | --- | --- | --- | --- | --- | --- | --- | --- | --- |
| MPR_144 | 2337.62 | 1914.17 | 1241.46 | 64.86 | 92.35 | 17.43 | 80.56 | 63.33 | 33.6 |
| MPR_145 | 1876.91 | 1582.26 | 1007.55 | 63.68 | 92.78 | 14.17 | 80.41 | 58.62 | 24.66 |
| MPR_146 | 2032.54 | 1727.97 | 1086.58 | 62.88 | 93.07 | 15.42 | 81.48 | 61.43 | 28.07 |
| MPR_147 | 1806.45 | 1512.96 | 978.18 | 64.65 | 92.57 | 13.73 | 78.81 | 56.5 | 23.14 |
| MPR_148 | 1768.15 | 1483.43 | 932.74 | 62.88 | 92.4 | 13.13 | 78.11 | 54.66 | 21.53 |
| MPR_150 | 1743.35 | 1441.25 | 923.41 | 64.07 | 92.34 | 12.96 | 78.3 | 54.54 | 20.84 |
| MPR_151 | 2701.42 | 2191.66 | 1433.75 | 65.42 | 89.81 | 20.23 | 75.61 | 62.11 | 38.95 |
| MPR_152 | 1919.55 | 1600.79 | 1023.62 | 63.94 | 92.56 | 14.33 | 79.85 | 58.26 | 25.09 |
| MPR_153 | 1693.34 | 1168.52 | 886.69 | 75.88 | 92.43 | 12.46 | 77.57 | 52.94 | 19.32 |
| MPR_154 | 1794.6 | 1487.4 | 952.05 | 64.01 | 92.56 | 13.39 | 79 | 55.85 | 22.34 |
| MPR_155 | 1826.53 | 1519.08 | 975.67 | 64.23 | 92.85 | 13.78 | 79.39 | 56.78 | 23.12 |
| MPR_156 | 1917.39 | 1575.76 | 1006.49 | 63.87 | 92.66 | 14.08 | 80.12 | 58.21 | 24.39 |
| MPR_159 | 2617.35 | 2107.7 | 1332.53 | 63.22 | 93.51 | 18.77 | 85.36 | 70.04 | 38.37 |
| MPR_161 | 2273.23 | 1845.35 | 1203.41 | 65.21 | 93.2 | 16.87 | 83.49 | 65.64 | 32.55 |
| MPR_162 | 1780.77 | 1468.44 | 944.41 | 64.31 | 92.51 | 13.25 | 78.41 | 55.21 | 21.76 |
| MPR_163 | 3142.98 | 2546.46 | 1664.81 | 65.38 | 91.02 | 23.66 | 76.73 | 64.45 | 44.63 |
| MPR_164 | 3364.92 | 2720.38 | 1764.14 | 64.85 | 93.08 | 24.93 | 82.94 | 70.81 | 48.66 |
| MPR_165 | 1909.51 | 1556.6 | 984.97 | 63.28 | 92.83 | 13.86 | 79.61 | 57.4 | 23.76 |
| MPR_166 | 2092.19 | 1735.58 | 1104.78 | 63.65 | 93 | 15.6 | 81.16 | 61.43 | 28.8 |
| MPR_167 | 1701.36 | 1417.83 | 899.35 | 63.43 | 92.45 | 12.68 | 77.93 | 53.64 | 19.93 |
| MPR_168 | 2201.33 | 1781.88 | 1137.42 | 63.83 | 93.06 | 16.01 | 82.44 | 63.49 | 29.89 |
