# Supplementary material for: The genomic basis of environmental adaptation in house mice
Source: PLoS Genet. 2018 Sep 24;14(9):e1007672. doi: 10.1371/journal.pgen.1007672 (PMC6171964; doi:10.1371/journal.pgen.1007672)
Supplement: S10 Table — (DOCX) [file pgen.1007672.s010.docx]

Supplementary Table 10. Pairwise differentiation (*F_st_*) and geographic distance (km) among surveyed populations.

| Population | Population | Geographic Distance (km) | *F_st_* Exome | *F_st_* Genome |
| --- | --- | --- | --- | --- |
| Florida | Georgia | 508.14 | 0.089 | 0.098 |
| Florida | Virginia | 1005.76 | 0.079 | 0.073 |
| Florida | Pennsylvania | 1367.60 | 0.065 | 0.068 |
| Florida | New Hampshire/Vermont | 1845.74 | 0.128 | 0.127 |
| Georgia | Virginia | 667.89 | 0.085 | 0.090 |
| Georgia | Pennsylvania | 1002.11 | 0.141 | 0.155 |
| Georgia | New Hampshire/Vermont | 1478.59 | 0.206 | 0.216 |
| Virginia | Pennsylvania | 361.89 | 0.120 | 0.121 |
| Virginia | New Hampshire/Vermont | 840.95 | 0.180 | 0.174 |
| Pennsylvania | New Hampshire/Vermont | 481.29 | 0.164 | 0.164 |
