# Supplementary material for: The genomic basis of environmental adaptation in house mice
Source: PLoS Genet. 2018 Sep 24;14(9):e1007672. doi: 10.1371/journal.pgen.1007672 (PMC6171964; doi:10.1371/journal.pgen.1007672)
Supplement: S11 Table — (DOCX) [file pgen.1007672.s011.docx]

Supplementary Table 11. The number of candidate genes identified in all three methods in the exome that show evidence of a drop-off of the signal of selection within a given window upstream and downstream of the primary candidate SNP (see Methods for details).

| Window in which data first available (kb) | Window in which max LFMM z-score first drops below cut-off (kb) | # of candidate genes that drop below cut-off \|z\| >=3 in this window upstream | # of candidate genes that drop below cut-off \|z\| >=3 in this window downstream | # of candidate genes that drop below cut-off \|z\| >= 2 in this window upstream | # of candidate genes that drop below cut-off \|z\| >=2 in this window downstream |
| --- | --- | --- | --- | --- | --- |
| 2 | 2 | 43 | 45 | 25 | 29 |
| 2 | 4 | 7 | 1 | 9 | 6 |
| 2 | 6 | 3 | 3 | 8 | 3 |
| 2 | 8 | 1 | 1 | 1 | 3 |
| 2 | 10 | 0 | 0 | 2 | 1 |
| 2 | 12 | 1 | 1 | 2 | 1 |
| 2 | 14 | 1 | 0 | 2 | 0 |
| 2 | 18 | 0 | 0 | 1 | 0 |
| 2 | 20 | 1 | 0 | 2 | 0 |
| 2 | 22+ | 1 | 4 | 8 | 12 |
| 4 | 4 | 21 | 18 | 18 | 14 |
| 4 | 6 | 0 | 4 | 0 | 3 |
| 4 | 8 | 0 | 1 | 1 | 2 |
| 4 | 10 | 0 | 0 | 1 | 0 |
| 4 | 12 | 0 | 0 | 0 | 0 |
| 4 | 14 | 1 | 0 | 1 | 1 |
| 4 | 16 | 0 | 0 | 1 | 0 |
| 4 | 22 | 0 | 0 | 0 | 1 |
| 4 | 22+ | 0 | 0 | 0 | 2 |
| 6 | 6 | 8 | 12 | 7 | 10 |
| 6 | 8 | 0 | 1 | 0 | 1 |
| 6 | 14 | 0 | 0 | 0 | 1 |
| 6 | 22+ | 0 | 0 | 1 | 1 |
| 8 | 8 | 7 | 9 | 7 | 7 |
| 8 | 10 | 0 | 0 | 0 | 1 |
| 8 | 22+ | 0 | 0 | 0 | 1 |

Supplementary Table 11, cont’d. The number of candidate genes identified in all three methods in the exome that show evidence of a drop-off of the signal of selection within a given window upstream and downstream of the primary candidate SNP.

| Window in which data first available (kb) | Window in which max LFMM z-score first drops below cut-off (kb) | # of candidate genes that drop below cut-off \|z\| >=3 in this window upstream | # of candidate genes that drop below cut-off \|z\| >=3 in this window downstream | # of candidate genes that drop below cut-off \|z\| >= 2 in this window upstream | # of candidate genes that drop below cut-off \|z\| >=2 in this window downstream |
| --- | --- | --- | --- | --- | --- |
| 10 | 10 | 3 | 4 | 0 | 3 |
| 10 | 12 | 0 | 0 | 1 | 1 |
| 12 | 12 | 3 | 4 | 1 | 3 |
| 12 | 22+ | 0 | 0 | 1 | 1 |
| 14 | 14 | 1 | 1 | 1 | 0 |
| 14 | 18 | 1 | 0 | 0 | 0 |
| 14 | 20 | 1 | 0 | 1 | 0 |
| 14 | 22 | 0 | 1 | 0 | 1 |
| 14 | 22+ | 0 | 0 | 1 | 1 |
| 16 | 16 | 5 | 1 | 3 | 1 |
| 16 | 20 | 0 | 0 | 1 | 0 |
| 16 | 22+ | 2 | 0 | 3 | 0 |
| 18 | 18 | 1 | 1 | 1 | 1 |
| 20 | 20 | 5 | 3 | 5 | 2 |
| 20 | 22+ | 0 | 0 | 0 | 1 |
| 22 | 22 | 2 | 0 | 2 | 0 |
| 22+ | 22+ | 15 | 21 | 15 | 21 |
