# Supplementary material for: The genomic basis of environmental adaptation in house mice
Source: PLoS Genet. 2018 Sep 24;14(9):e1007672. doi: 10.1371/journal.pgen.1007672 (PMC6171964; doi:10.1371/journal.pgen.1007672)
Supplement: S12 Table — (DOCX) [file pgen.1007672.s012.docx]

Supplementary Table 12. The primary annotation for candidate SNPs identified via different methods and in the full dataset.

| Analysis | Missense / Nonsense | UTR | Synonymous | Other Coding | Intron/ Splice Site | Upstream/ Downstream | Intergenic |
| --- | --- | --- | --- | --- | --- | --- | --- |
| Top 5% correlation coefficient and slope | 69 | 218 | 112 | 7 | 307 | 12 | 0 |
| Top 2.5% correlation coefficient | 284 | 802 | 465 | 98 | 1290 | 62 | 7 |
| LFMM \|z-score\| ≥ 3 | 113 | 386 | 211 | 29 | 542 | 26 | 0 |
| LFMM \|z-score\| ≥ 2 | 1185 | 4129 | 2487 | 430 | 5880 | 272 | 36 |
| All SNPs | 25,815 | 79,469 | 45,438 | 9,544 | 114,703 | 5,622 | 770 |
