# Supplementary material for: The genomic basis of environmental adaptation in house mice
Source: PLoS Genet. 2018 Sep 24;14(9):e1007672. doi: 10.1371/journal.pgen.1007672 (PMC6171964; doi:10.1371/journal.pgen.1007672)
Supplement: S13 Table — (DOCX) [file pgen.1007672.s013.docx]

Supplementary Table 13. Yields of data obtained via HiSeq2000 sequencing of genomic libraries.

| Individual | # of Bases Mapped | # of Bases with ≥1 Read | Avg Depth at Sites with ≥1 Read | Avg Depth across non-N genome |
| --- | --- | --- | --- | --- |
| MPR_108 | 8,501,854,237 | 2,173,462,537 | 3.91 | 3.21 |
| MPR_110 | 5,409,238,053 | 1,949,915,720 | 2.77 | 2.04 |
| MPR_112 | 4,314,336,169 | 1,832,597,368 | 2.35 | 1.63 |
| MPR_113 | 1,937,736,926 | 784,003,117 | 2.47 | 0.73 |
| MPR_114 | 3,997,872,754 | 1,794,830,851 | 2.23 | 1.51 |
| MPR_115 | 6,641,445,320 | 2,093,611,275 | 3.17 | 2.51 |
| MPR_116 | 8,324,766,198 | 2,090,709,071 | 3.98 | 3.14 |
| MPR_118 | 6,643,605,271 | 2,063,262,811 | 3.22 | 2.51 |
| MPR_120 | 7,110,675,651 | 2,072,198,011 | 3.43 | 2.69 |
| MPR_121 | 7,174,229,045 | 1,815,981,534 | 3.95 | 2.71 |
| MPR_123 | 4,193,778,874 | 1,566,679,615 | 2.68 | 1.58 |
| MPR_124 | 8,492,706,773 | 2,160,876,465 | 3.93 | 3.21 |
| MPR_125 | 6,079,701,117 | 2,032,512,902 | 2.99 | 2.30 |
| MPR_126 | 5,983,606,159 | 1,715,238,264 | 3.49 | 2.26 |
| MPR_128 | 6,154,869,309 | 2,039,808,843 | 3.02 | 2.32 |
| MPR_129 | 5,753,455,638 | 1,992,283,530 | 2.89 | 2.17 |
| MPR_130 | 6,640,864,786 | 2,068,990,044 | 3.21 | 2.51 |
| MPR_131 | 6,305,898,079 | 2,033,125,384 | 3.10 | 2.38 |
| MPR_132 | 6,257,617,293 | 1,839,229,607 | 3.40 | 2.36 |
| MPR_133 | 7,176,329,751 | 2,098,558,404 | 3.42 | 2.71 |
| MPR_134 | 6,611,379,661 | 2,095,765,572 | 3.15 | 2.50 |
| MPR_135 | 6,166,665,875 | 2,016,051,495 | 3.06 | 2.33 |
| MPR_137 | 3,759,304,010 | 1,731,565,296 | 2.17 | 1.42 |
| MPR_138 | 6,481,446,442 | 2,068,100,954 | 3.13 | 2.45 |
| MPR_140 | 8,171,915,661 | 2,067,685,113 | 3.95 | 3.09 |
| MPR_141 | 5,884,366,119 | 2,033,400,570 | 2.89 | 2.22 |
| MPR_142 | 6,067,772,483 | 2,062,215,455 | 2.94 | 2.29 |
| MPR_143 | 8,963,214,249 | 2,183,854,970 | 4.10 | 3.39 |
| MPR_144 | 6,321,560,671 | 2,067,100,708 | 3.06 | 2.39 |
| MPR_145 | 7,120,005,060 | 2,129,010,735 | 3.34 | 2.69 |
| MPR_146 | 7,723,229,207 | 2,136,614,664 | 3.61 | 2.92 |
| MPR_147 | 6,900,059,140 | 2,030,606,707 | 3.40 | 2.61 |
| MPR_148 | 6,839,385,009 | 2,085,401,757 | 3.28 | 2.58 |
| MPR_150 | 7,169,175,470 | 2,128,413,987 | 3.37 | 2.71 |
| MPR_151 | 6,588,830,799 | 2,087,823,407 | 3.16 | 2.49 |
| MPR_152 | 8,461,346,394 | 2,019,445,653 | 4.19 | 3.20 |
| MPR_153 | 9,679,910,220 | 2,194,310,309 | 4.41 | 3.66 |
| MPR_154 | 7,246,288,403 | 2,132,762,258 | 3.40 | 2.74 |
| MPR_155 | 8,565,922,274 | 2,109,380,093 | 4.06 | 3.24 |
| MPR_156 | 4,348,945,648 | 1,860,188,551 | 2.34 | 1.64 |
| MPR_159 | 7,234,113,780 | 2,041,456,625 | 3.54 | 2.73 |
| MPR_161 | 6,931,900,875 | 2,102,690,360 | 3.30 | 2.62 |
| MPR_162 | 8,769,238,385 | 2,161,098,213 | 4.06 | 3.31 |
| MPR_163 | 5,678,875,087 | 1,501,688,370 | 3.78 | 2.14 |
| MPR_164 | 5,581,947,559 | 1,698,403,312 | 3.29 | 2.11 |
| MPR_165 | 5,315,015,332 | 1,990,836,245 | 2.67 | 2.01 |

Supplementary Table 13, cont’d. Yields of data obtained via HiSeq2000 sequencing of genomic libraries.

| Individual | # of Bases Mapped | # of Bases with ≥1 Read | Avg Depth at Sites with ≥1 Read | Avg Depth across non-N genome |
| --- | --- | --- | --- | --- |
| MPR_166 | 7,105,744,997 | 2,056,359,214 | 3.46 | 2.68 |
| MPR_167 | 4,619,603,204 | 1,566,742,628 | 2.95 | 1.74 |
| MPR_168 | 8,021,404,504 | 2,149,666,086 | 3.73 | 3.03 |
| MPR_169 | 7,394,193,794 | 2,125,608,360 | 3.48 | 2.79 |
