# Supplementary material for: The genomic basis of environmental adaptation in house mice
Source: PLoS Genet. 2018 Sep 24;14(9):e1007672. doi: 10.1371/journal.pgen.1007672 (PMC6171964; doi:10.1371/journal.pgen.1007672)
Supplement: S14 Table — (DOCX) [file pgen.1007672.s014.docx]

Supplementary Table 14. Number of candidate windows identified in the genome that fall in or near genes and/or near putative promoters.

| Window Analysis | # of candidate windows within ±5 kb of a gene | # of candidate windows that are not within ±5 kb of a gene | # of candidate windows within ±500 bp of a putative promoter | # of candidate windows that are near a gene and a promoter |
| --- | --- | --- | --- | --- |
| 1 kb, 500 bp step | 4995 | 4722 | 888 | 687 |
| 1.5 kb, 750 bp step | 2965 | 2962 | 588 | 455 |
| 2.5 kb, 2.5 kb step | 1057 | 1092 | 233 | 178 |
