# Supplementary material for: The genomic basis of environmental adaptation in house mice
Source: PLoS Genet. 2018 Sep 24;14(9):e1007672. doi: 10.1371/journal.pgen.1007672 (PMC6171964; doi:10.1371/journal.pgen.1007672)
Supplement: S15 Table — (DOCX) [file pgen.1007672.s015.docx]

Supplementary Table 15. The number of genes with evidence of differential expression (DE) or allele specific expression (ASE) in lab raised mice derived from wild populations in New York and Florida.

| Tissue | Generation | # of genes with evidence of DE  *P_a_*_dj_ < 0.05 | # of genes with evidence of DE  *P_a_*_dj_ < 0.10 | # of genes with evidence of ASE  *P_a_*_dj_ < 0.05 | # of genes with evidence of ASE  *P_a_*_dj_ < 0.10 |
| --- | --- | --- | --- | --- | --- |
| Fat | N_1_ | 3514 | 4796 | 1515 | 1645 |
| Hypothalamus | N_1_ | 77 | 111 | 1459 | 1647 |
| Liver | N_1_ | 17 | 20 | 1386 | 1485 |
| Liver | N_2_ | 112 | 163 | 1253 | 1344 |
