# Supplementary material for: The genomic basis of environmental adaptation in house mice
Source: PLoS Genet. 2018 Sep 24;14(9):e1007672. doi: 10.1371/journal.pgen.1007672 (PMC6171964; doi:10.1371/journal.pgen.1007672)
Supplement: S16 Table — (DOCX) [file pgen.1007672.s016.docx]

Supplementary Table 16. Genes identified as candidates in the genome analyses that also show evidence of differential expression (DE) and allele specific expression (ASE) in the same tissue. All tissues come from N_1_ mice unless noted. F indicates fat, L indicates liver, and H indicates hypothalamus.

|  |  |  |  | Function, Phenotype, or QTL^*^ related to: | | | | | |
| --- | --- | --- | --- | --- | --- | --- | --- | --- | --- |
| Gene Name | Chr:Start (bp) | Tissue w/ evidence of DE | Tissue w/  evidence of  ASE | Body Size | Fat/ Obesity | Circadian Rhythm | Immunity | Blood Chemistry/ Diabetes | Nesting |
| *Col3a1^*^* | 1:45,311,538 | F | F | Y | N | N | Y | N | N |
| *Steap3* | 1:120,190,757 | F | F | Y | Y | N | Y | Y | N |
| *Dpt* | 1:164,796,644 | F | F | Y | Y | N | Y | Y | N |
| *Capn2* | 1:182,467,260 | F | F, H | Y | Y | N | Y | N | N |
| *Plxdc2* | 2:16,356,304 | F | F | N | N | N | N | N | N |
| *Swi5* | 2:32,278,816 | F | F | Y | N | N | Y | Y | N |
| *Apmap* | 2:150,583,080 | F | F | Y | Y | Y | Y | Y | N |
| *Acad9* | 3:36,065,979 | F | F | Y | Y | N | N | Y | N |
| *Slc25a51* | 4:45,395,923 | L | L N_2_ | Y | N | N | Y | N | N |
| *Jak1* | 4:101,152,367 | F | F | Y | N | N | Y | Y | N |
| *Dhrs3* | 4:144,892,827 | F | F, L N_1_ & N_2_ | Y | N | N | N | N | N |
| *Cyp51* | 5:4,081,145 | F | F, L N_1_ & N_2_ | N | N | N | Y | Y | N |
| *Gnai1* | 5:18,265,135 | F | F | N | N | N | N | N | N |
| *Urad* | 5:147,314,984 | L N_2_ | L N_1_ | N | N | N | N | Y | N |
| *Pomp^*^* | 5:147,860,461 | F | ALL | N | N | N | N | Y | N |
| *Cav1* | 6:17,306,335 | F | F | Y | Y | N | Y | Y | N |
| *Akr1b8* | 6:34,354,119 | F | F | Y | Y | N | N | Y | N |
| *Cyfip1* | 7:55,841,745 | F | F | Y | Y | N | Y | N | N |
| *Serpinh1* | 7:99,345,376 | F | F | Y | N | N | Y | Y | N |
| *Col4a2* | 8:11,312,805 | F | F | N | N | N | N | Y | N |
| *Gas6* | 8:13,465,374 | F | F, H | N | N | N | Y | Y | N |
| *Eif4ebp1* | 8:27,260,329 | F | F, L N_1_ & N_2_ | Y | Y | N | Y | Y | N |
| *Itfg1* | 8:85,717,578 | F | F, H | Y | N | N | Y | N | N |
| *Gcsh* | 8:116,981,810 | F | ALL | Y | Y | N | N | N | N |

^*^Also identified in all exome analyses, see Table 1.

Supplementary Table 16, cont’d. Genes identified as candidates in the genome analyses that also show evidence of differential expression (DE) and allele specific expression (ASE) in the same tissue. All tissues come from N_1_ mice unless noted. F indicates fat, L indicates liver, and H indicates hypothalamus.

|  |  |  |  | Phenotype^*^ or QTL^*^ related to: | | | | | |
| --- | --- | --- | --- | --- | --- | --- | --- | --- | --- |
| Gene Name | Chr:Start (bp) | Tissue w/ evidence of DE | Tissue w/  evidence of ASE | Body Size | Fat/ Obesity | Circadian Rhythm | Immunity | Blood Chemistry/ Diabetes | Nesting |
| *Sc5d* | 9:42,254,177 | F | F, L N_1_ & N_2_ | Y | Y | N | Y | Y | N |
| *Fbxo22^*^* | 9:55,208,925 | F | F, L N_1_ | Y | Y | N | Y | N | N |
| *Nrg4^*^* | 9:55,220,222 | F | F | Y | Y | N | Y | N | N |
| *Lama4* | 10:38,965,515 | F | F | Y | Y | N | Y | N | N |
| *Zwint* | 10:72,654,845 | F | ALL | Y | Y | N | Y | N | N |
| *Col6a2* | 10:76,595,762 | F, L N2 | F | Y | Y | N | Y | Y | N |
| *Dram1* | 10:88,322,804 | F | F | Y | Y | N | Y | Y | N |
| *Dcn* | 10:97,479,500 | F | F, H, L N_1_ | Y | Y | N | Y | Y | N |
| *Ogdh* | 11:6,291,633 | F | F | Y | Y | N | Y | N | N |
| *Sptbn1* | 11:30,099,395 | F | F, H | Y | Y | N | Y | Y | N |
| *Dusp3* | 11:101,971,143 | F | F, H | Y | N | N | Y | Y | N |
| *Auh* | 13:52,835,119 | F | F, H, L N_1_ | N | N | N | N | N | N |
| *Sfxn1* | 13:54,071,845 | L N2 | F, L N_1_ & N_2_ | N | N | N | N | N | N |
| *Arhgef3* | 14:27,238,039 | F | F, L N_2_ | N | Y | N | N | N | N |
| *Tuba1a* | 15:98,949,841 | F | F, H | Y | Y | N | Y | N | Y |
| *Tuba1c* | 15:99,029,891 | F | F, L N2 | Y | Y | N | Y | N | N |
| *Itgb5* | 16:33,829,665 | F | F, L N_1_ & N_2_ | Y | N | N | N | N | N |
| *Cyp39a1* | 17:43,667,425 | L N2 | L N_2_ | N | N | N | Y | Y | N |
| *Ermp1* | 19:29,608,214 | F | F | Y | N | N | N | N | N |

^*^Also identified in all exome analyses, see Table 1.
