# Supplementary material for: The genomic basis of environmental adaptation in house mice
Source: PLoS Genet. 2018 Sep 24;14(9):e1007672. doi: 10.1371/journal.pgen.1007672 (PMC6171964; doi:10.1371/journal.pgen.1007672)
Supplement: S17 Table — (DOCX) [file pgen.1007672.s017.docx]

Supplementary Table 17. Overlap between genes identified as candidates for environmental adaptation in humans [60-62] and in our study.

| Human symbol | Mouse orthologue | Mouse symbol | Identified as candidate |
| --- | --- | --- | --- |
| Ace | 1-to-1 | Ace | Null |
| Adra2b | 1-to-1 | Adra2b | Null |
| Bak1 | 1-to-1 | Bak1 | Null |
| Cd36 | 1-to-1 | Cd36 | Null |
| Cd40 | 1-to-1 | Cd40 | LFMM Z-score ≥2 |
| Cetp | No orthologue |  | Null |
| Clock | 1-to-1 | Clock | LFMM Z-score ≥2 |
| Ctnnb1 | 1-to-1 | Ctnnb1 | Null |
| Drd2 | 1-to-1 | Drd2 | Null |
| Dscr1 | 1-to-1 | Rcan1 | Null |
| Egfr | 1-to-1 | Egfr | LFMM Z-score ≥2 |
| Ephx2 | 1-to-1 | Ephx2 | LFMM Z-score ≥2, Top 2.5% Correlation Coefficient |
| Fabp2 | 1-to-1 | Fabp2 | Null |
| Fgf2 | 1-to-1 | Fgf2 | Null |
| Herc2 | 1-to-1 | Herc2 | LFMM Z-score ≥2 |
| Hla-c | Many-to-many |  | Null |
| Hla-dqa1 | 1-to-many | H2-aa | Null |
| Ipf1 | 1-to-1 | Pdx1 | Null |
| Lepr | 1-to-1 | Lepr | LFMM Z-score ≥2 |
| Lpa | 1-to-many | Plg | Null |
| Mapk1 | 1-to-1 | Mapk1 | LFMM Z-score ≥2 |
| Mapk14 | 1-to-1 | Mapk14 | LFMM Z-score ≥3, Top 2.5% Correlation Coefficient |
| Mef2a | 1-to-1 | Mef2a | Null |
| Meox2 | 1-to-1 | Meox2 | Null |
| Mmrn1 | 1-to-1 | Mmrn1 | Null |
| Nppa | 1-to-1 | Nppa | LFMM Z-score ≥2 |
| Nudt6 | 1-to-1 | Nudt6 | Null |
| Oca2 | 1-to-1 | Oca2 | LFMM Z-score ≥2 |
| Pcdh18 | 1-to-1 | Pcdh18 | Null |
| Pcsk1 | 1-to-1 | Pcsk1 | LFMM Z-score ≥2 |
| Pik3cb | 1-to-1 | Pik3cb | LFMM Z-score ≥2, Top 2.5% Correlation Coefficient |
| Pon1 | 1-to-1 | Pon1 | LFMM Z-score ≥2 |
| Ppargc1a | 1-to-1 | Ppargc1a | LFMM Z-score ≥2 |
| Ptger4 | 1-to-1 | Ptger4 | Null |
| Ptk2b | 1-to-1 | Ptk2b | Null |

Supplementary Table 17, cont’d. Overlap between genes identified as candidates for environmental adaptation in humans (59-61) and in our study.

| Human symbol | Mouse orthologue | Mouse symbol | Identified as candidate |
| --- | --- | --- | --- |
| Rptor | 1-to-1 | Rptor | Genome Window |
| Scarb2 | 1-to-1 | Scarb2 | LFMM Z-score ≥2 |
| Sdk1 | 1-to-1 | Sdk1 | Top 2.5% Correlation Coefficient |
| Slc45a2 | 1-to-1 | Slc45a2 | Null |
| Soat1 | 1-to-1 | Soat1 | Null |
| Sod1 | 1-to-1 | Sod1 | Null |
| Tcf1 | 1-to-1 | Hnf1a | LFMM Z-score ≥2 |
| Tcf7l2 | 1-to-1 | Tcf7l2 | Null |
| Tnxb | 1-to-1 | Tnxb | LFMM Z-score ≥2, Top 2.5% Correlation Coefficient |
| Ucp1 | 1-to-1 | Ucp1 | Null |
| Ucp2 | 1-to-1 | Ucp2 | Null |
| Ucp3 | 1-to-1 | Ucp3 | LFMM Z-score ≥2 |
