# Supplementary material for: The genomic basis of environmental adaptation in house mice
Source: PLoS Genet. 2018 Sep 24;14(9):e1007672. doi: 10.1371/journal.pgen.1007672 (PMC6171964; doi:10.1371/journal.pgen.1007672)
Supplement: S18 Table — (DOCX) [file pgen.1007672.s018.docx]

Supplementary Table 18. Loading matrix and values for the first four principal components summarizing climate variables

| Factor | PC1 | PC2 | PC3 | PC4 |
| --- | --- | --- | --- | --- |
| BIO1 | 0.988 | 0.133 | 0.010 | 0.080 |
| BIO2 | 0.511 | -0.276 | 0.810 | 0.079 |
| BIO3 | 0.986 | -0.132 | 0.104 | 0.003 |
| BIO4 | -1.000 | 0.011 | 0.018 | -0.016 |
| BIO5 | 0.933 | 0.331 | 0.059 | 0.130 |
| BIO6 | 0.983 | 0.160 | -0.068 | 0.064 |
| BIO7 | -0.989 | -0.077 | 0.125 | -0.032 |
| BIO8 | 0.245 | -0.698 | -0.367 | 0.564 |
| BIO9 | 0.925 | 0.290 | 0.155 | -0.191 |
| BIO10 | 0.952 | 0.274 | 0.009 | 0.133 |
| BIO11 | 0.993 | 0.103 | -0.007 | 0.065 |
| BIO12 | 0.898 | 0.307 | -0.247 | -0.195 |
| BIO13 | 0.953 | -0.212 | -0.066 | -0.204 |
| BIO14 | 0.010 | 0.993 | -0.039 | 0.110 |
| BIO15 | 0.759 | -0.617 | -0.002 | -0.207 |
| BIO16 | 0.946 | -0.227 | -0.117 | -0.200 |
| BIO17 | 0.072 | 0.962 | -0.215 | 0.150 |
| BIO18 | 0.861 | -0.457 | -0.218 | -0.049 |
| BIO19 | 0.588 | 0.767 | 0.079 | -0.244 |
| NSWRS | 0.960 | 0.092 | -0.217 | 0.150 |
| Specific Humidity | 0.836 | -0.533 | -0.105 | -0.071 |
| Relative Humidity | 0.975 | -0.104 | 0.165 | -0.110 |
| % Sunshine | 0.780 | 0.102 | 0.338 | 0.517 |
| **Eigenvalue** | 16.288 | 4.504 | 1.230 | 0.978 |
| **% Variance Explained** | 70.818 | 19.583 | 5.348 | 4.251 |
| **Florida** | 5.920 | -2.114 | -0.226 | -0.120 |
| **Georgia** | 1.609 | 2.821 | 0.868 | -0.805 |
| **Virginia** | -0.503 | 0.771 | 0.250 | 1.713 |
| **Pennsylvania** | -2.378 | 0.658 | -1.798 | -0.354 |
| **New Hampshire/Vermont** | -4.649 | -2.136 | 0.906 | -0.435 |
