# Supplementary material for: The genomic basis of environmental adaptation in house mice
Source: PLoS Genet. 2018 Sep 24;14(9):e1007672. doi: 10.1371/journal.pgen.1007672 (PMC6171964; doi:10.1371/journal.pgen.1007672)
Supplement: S19 Table — (DOCX) [file pgen.1007672.s019.docx]

Supplementary Table 19. Estimates of nucleotide diversity for the surveyed populations.

|  | Avg per site Watterson’s θ (%) | | Avg per site π (%) | |
| --- | --- | --- | --- | --- |
| Population | 10kb genomic windows^1^ | Intronic  sites in the exome^2^ | 10kb genomic windows^1^ | Intronic sites in the exome^2^ |
| Florida | 0.2850 | 0.2134 | 0.2963 | 0.2332 |
| Georgia | 0.1887 | 0.1563 | 0.2219 | 0.1847 |
| Virginia | 0.2142 | 0.1768 | 0.2437 | 0.1964 |
| Pennsylvania | 0.2140 | 0.1699 | 0.2492 | 0.1994 |
| New Hampshire/ Vermont | 0.1694 | 0.1388 | 0.2019 | 0.1657 |

^1^ 115,075 windows were included in the analysis, each with an average of 7,027 sites

^2^ 18,029,679 intronic sites were used to estimate nucleotide diversity from the exome data
