# Supplementary material for: The genomic basis of environmental adaptation in house mice
Source: PLoS Genet. 2018 Sep 24;14(9):e1007672. doi: 10.1371/journal.pgen.1007672 (PMC6171964; doi:10.1371/journal.pgen.1007672)

Supplementary Figure 1. Climate variables versus latitude for locations included in the transect. Two BioClim variables had negligible loading values for principal component 1 summarizing all climate variables and are not included in the plot (mean temperature of wettest quarter and precipitation of driest month). The inverse values for temperature seasonality and temperature annual range are plotted for ease of interpretation.


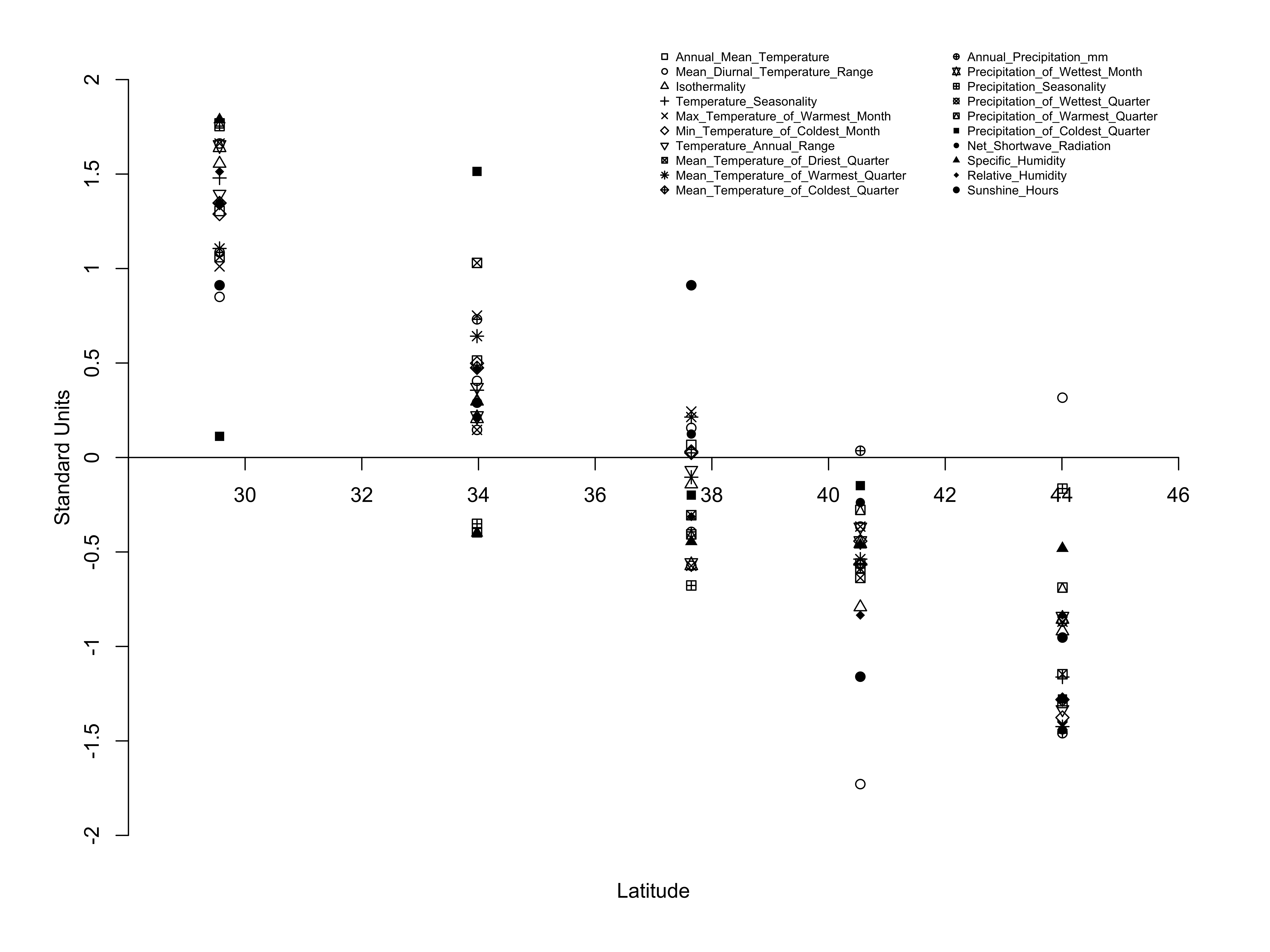

Supplement: S1 Fig — (DOCX) [file pgen.1007672.s020.docx]
