# Supplementary material for: The genomic basis of environmental adaptation in house mice
Source: PLoS Genet. 2018 Sep 24;14(9):e1007672. doi: 10.1371/journal.pgen.1007672 (PMC6171964; doi:10.1371/journal.pgen.1007672)
Supplement: S2 Fig — There is no evidence of a significant association between genetic distance and geographic distance (A) Geographic distance vs. pairwise Fst (B) genetic PCA. (DOCX) [file pgen.1007672.s021.docx]

Supplementary Figure 2. There is no evidence of a significant association between genetic distance and geographic distance. **(A)** Pairwise *F_st_* vs. geographic distance (Exome: Mantel test, Z= 1225.03, r = 0.12, *p* = 0.35, Reduced Major Axis (RMA) Regression with jackknife over populations: y= 9.7x10^-5^ x + 0.033, 95% CI for Intercept (-0.622, 0.198), 95% CI for slope (-0.000125, 0.00086); Genome: Mantel test, Z= 1235.65, r = 0.12, *p* = 0.36, RMA Regression with jackknife over populations: y = 9.9x10^-5^ x + 0.034, 95% CI for Intercept (-0.637, 0.195), 95% CI for slope (-0.000128, 0.00089). **(B)** Genetic Principle Component Analysis (PCA), PC1 (14.18% of variance) vs. PC2 (8.03% of variance)

**A**

­­­­­­

**B**
