# Supplementary material for: The genomic basis of environmental adaptation in house mice
Source: PLoS Genet. 2018 Sep 24;14(9):e1007672. doi: 10.1371/journal.pgen.1007672 (PMC6171964; doi:10.1371/journal.pgen.1007672)
Supplement: S3 Fig — The distribution of (A) R2 and (B) |slope| for the linear relationship between allele frequency and latitude for exomic SNPs. (DOCX) [file pgen.1007672.s022.docx]

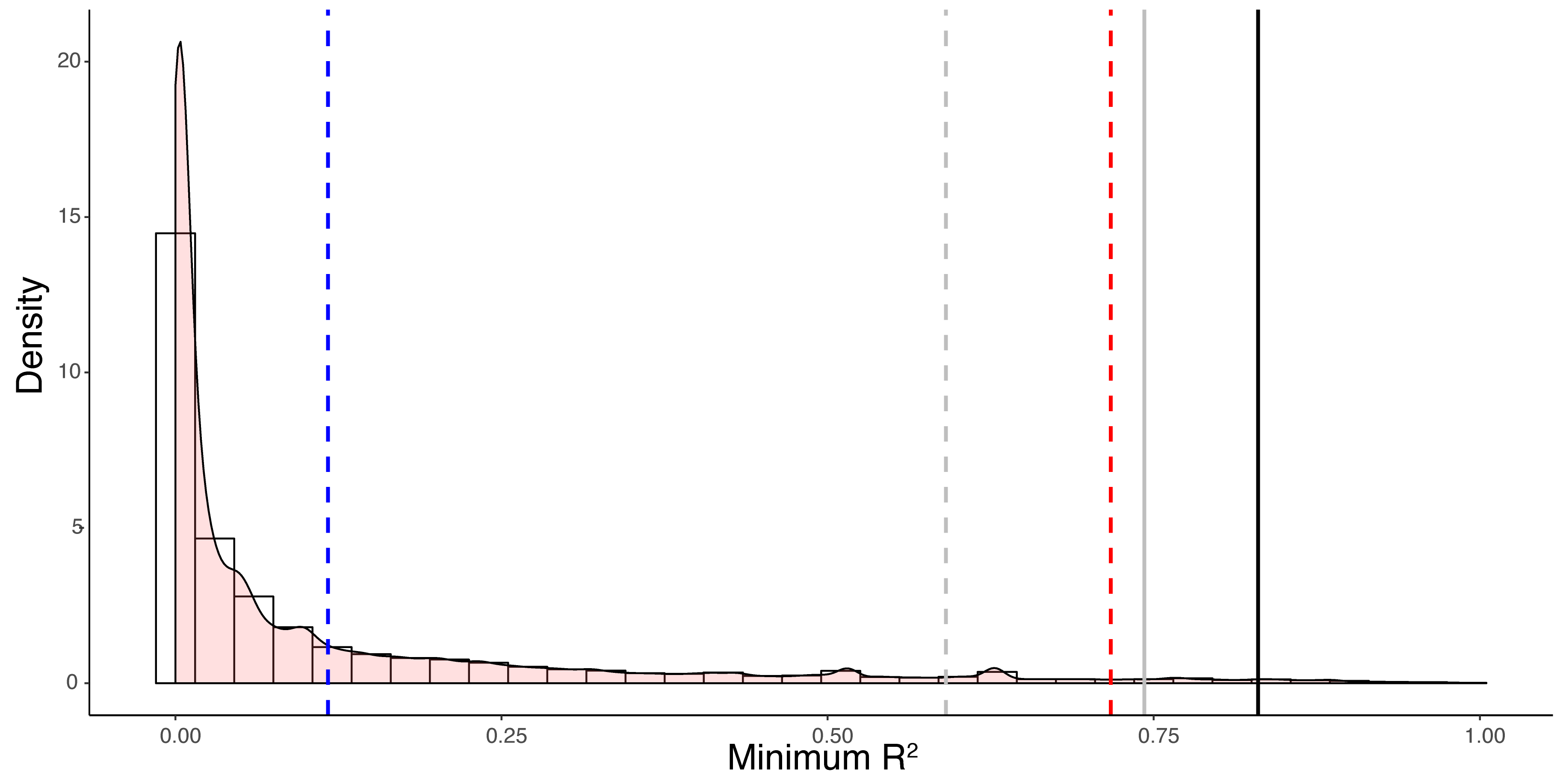
Supplementary Figure 3. The distribution of **(A)** R^2^ and **(B)** |slope| for the linear relationship between allele frequency and latitude for exomic SNPs. The data shown are the minimum values for each SNP when including all data or when any one population was dropped. For each plot, the blue dashed line indicates the mean, the dashed grey and red lines indicate the 95^th^ and 97.5^th^ percentile respectively. In **(A)** the solid grey line and the solid black line indicate 95^th^ and 97.5^th^ percentile cut-offs when all populations were included. In **(B)** the solid grey line indicates the 95^th^ percentile cut-off when all populations were included.

**A**


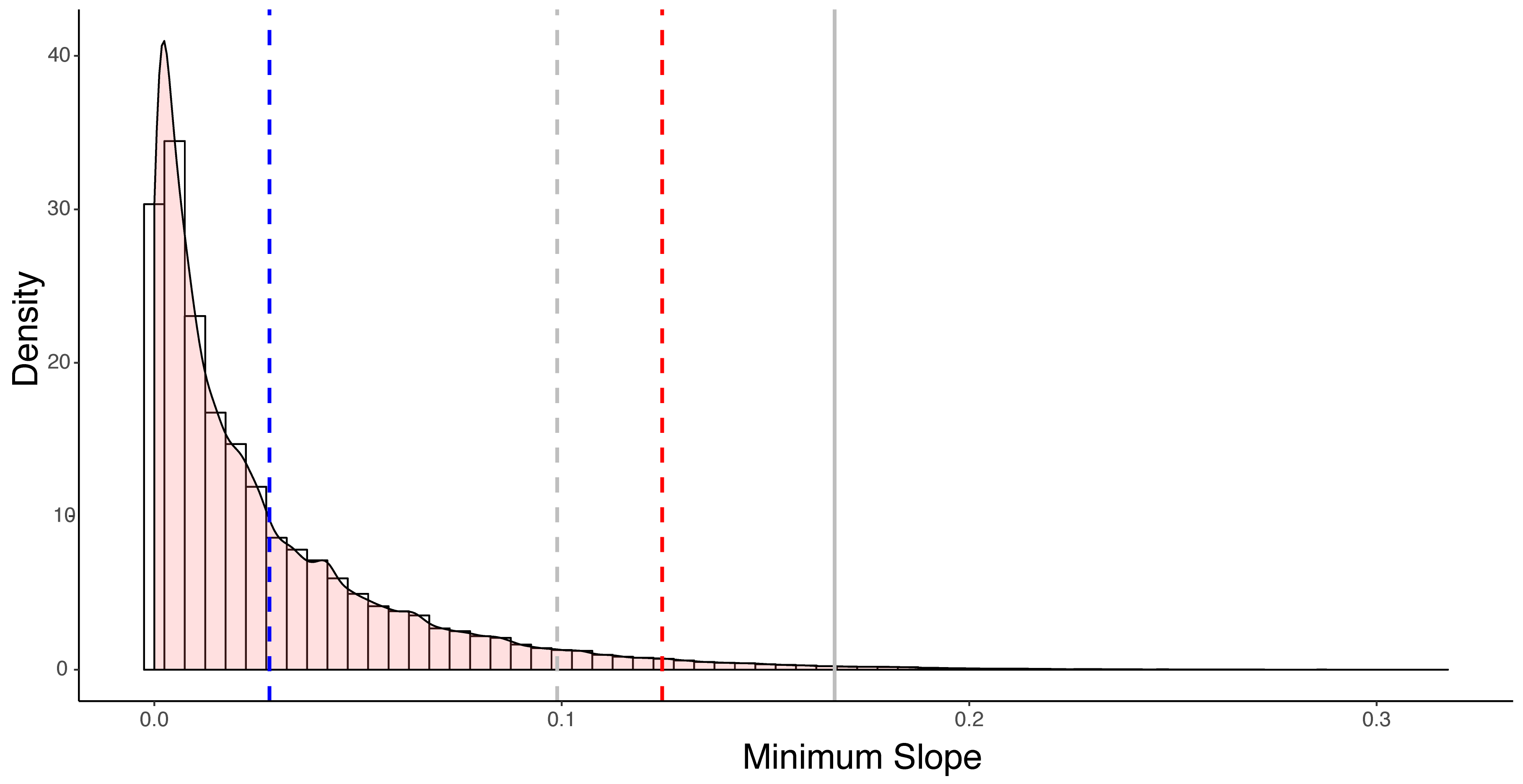


**B**
