# Supplementary material for: The genomic basis of environmental adaptation in house mice
Source: PLoS Genet. 2018 Sep 24;14(9):e1007672. doi: 10.1371/journal.pgen.1007672 (PMC6171964; doi:10.1371/journal.pgen.1007672)

Supplementary Figure 4. The correlation between allele frequency estimates from the exomic and genomic data (**A)** Allele frequency estimates given the entire sample of fifty individuals from common sites in the exome and genome data. The red line shows the regression line y = 0.97772 x -0.001387 (Pearson’s r =0.97, df = 242,136, *p* < 3 x 10^-16^); **(B)** Allele frequency estimates given individual populations derived from common sites in the exome and genome data. The red line shows the regression line y = 0.89270 x + 0.01901 (Pearson’s r=0.90, df = 989,907, *p* < 3 x 10^-16^).


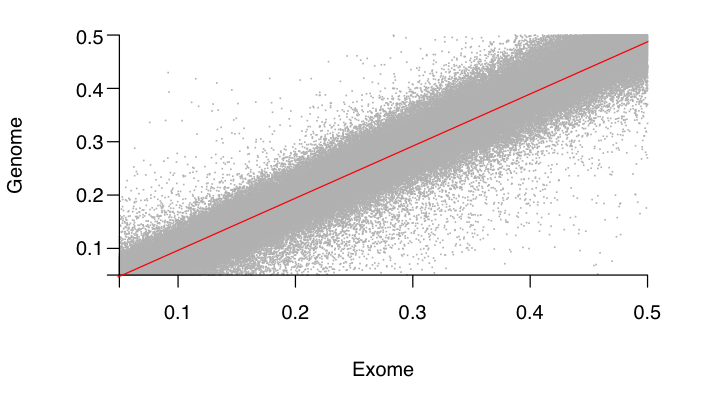


**A**

**B**


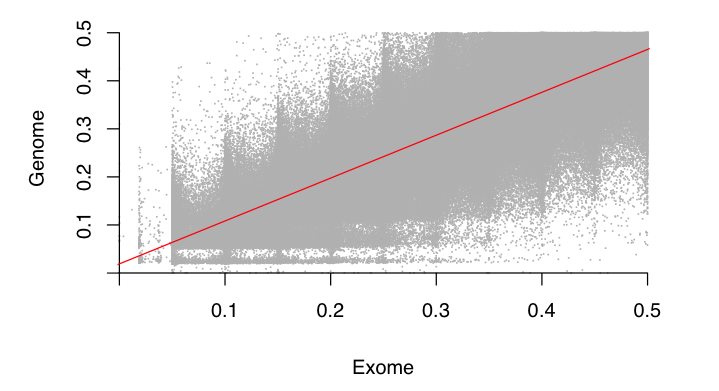

Supplement: S4 Fig — The correlation between allele frequency estimates from the exomic and genomic data (A) given the entire sample of 50 individuals (B) given individual populations. (DOCX) [file pgen.1007672.s023.docx]
