# Supplementary material for: The genomic basis of environmental adaptation in house mice
Source: PLoS Genet. 2018 Sep 24;14(9):e1007672. doi: 10.1371/journal.pgen.1007672 (PMC6171964; doi:10.1371/journal.pgen.1007672)
Supplement: S5 Fig — The first two principal components of variation in gene expression data from four tissues in male mice (A) hypothalamus N1 (B) liver N1 (C) liver N2 (D) Fat N1. (DOCX) [file pgen.1007672.s024.docx]

Supplementary Figure 5. The first two principal components of variation in gene expression data from four tissues in male mice. Blue dots represent mice from New York; red dots represent mice from Florida. The percent of variation explained by each component is given next to that axis. **(A)** hypothalamus (N_1_); **(B)** liver (N_1_); **(C)** liver (N_2_); **(D)** Fat (N_1_).
