# Supplementary material for: The genomic basis of environmental adaptation in house mice
Source: PLoS Genet. 2018 Sep 24;14(9):e1007672. doi: 10.1371/journal.pgen.1007672 (PMC6171964; doi:10.1371/journal.pgen.1007672)
Supplement: S6 Fig — The first two principal components of variation in gene expression data from the (A) liver of N2 male mice and (B) fat of N1 male mice after removal of outliers. (DOCX) [file pgen.1007672.s025.docx]

Supplementary Figure 6. The first two principal components of variation in gene expression data from the **(A)** liver of N_2_ male mice after removal of an outlier **(B)** fat of N_1_ male mice after removal of two outliers. Blue dots represent mice from New York; red dots represent mice from Florida.
