# Supplementary material for: The genomic basis of environmental adaptation in house mice
Source: PLoS Genet. 2018 Sep 24;14(9):e1007672. doi: 10.1371/journal.pgen.1007672 (PMC6171964; doi:10.1371/journal.pgen.1007672)
Supplement: S7 Fig — (DOCX) [file pgen.1007672.s026.docx]

Supplementary Figure 7. The distribution of *F_st_* values for all genes and for different sets of candidate genes in the exome.


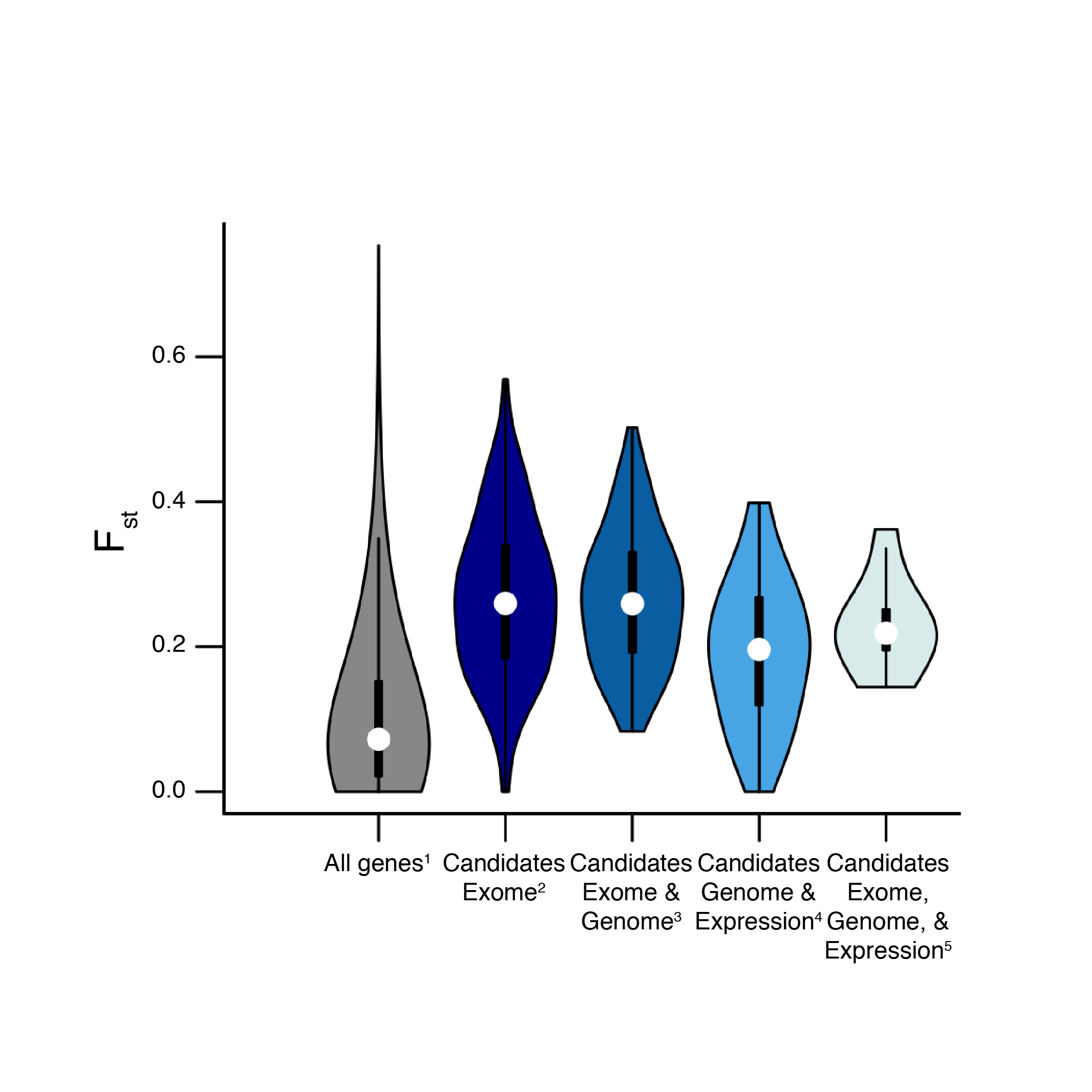


^1^ All genes for which *F_st_* could be estimated from the exome data (20,367 genes)

^2^ All of the 177 candidates identified by different methods in the exome for which *F_st_* could be estimated from the exome data (162 genes)

^3^All of the 127 candidates identified by different methods in the exome and in the genome for which *F_st_* could be estimated from the exome data (122 genes)

^4^All of the 43 candidates identified in the genome for which there was also evidence of *cis*-eQTL and for which *F_st_* could be estimated from the exome data (41 genes)

^5^All of the 10 candidates identified by the different methods in the exome, in the genome, and for which there was also differential expression and allele specific expression.
