# Supplementary material for: The genomic basis of environmental adaptation in house mice
Source: PLoS Genet. 2018 Sep 24;14(9):e1007672. doi: 10.1371/journal.pgen.1007672 (PMC6171964; doi:10.1371/journal.pgen.1007672)
Supplement: S8 Fig — Candidate gene Mc3r (A) Exome data show that allele frequencies for SNPs in Mc3r are highly correlated with latitude. (B) QTL and (C) knock-out mouse strains show that there are functional links between Mc3r and phenotypes that differ in our study among mice from different latitudes. (DOCX) [file pgen.1007672.s027.docx]

Supplementary Figure 8. Candidate gene *Mc3r*

**(A**) Exome data show that allele frequencies for SNPs in *Mc3r* are highly correlated with latitude. **(B**) QTL and (**C**) knock-out mouse strains show that there are functional links between *Mc3r* and phenotypes that differ in our study among mice from different latitudes [49, 50].
