# Supplementary material for: The genomic basis of environmental adaptation in house mice
Source: PLoS Genet. 2018 Sep 24;14(9):e1007672. doi: 10.1371/journal.pgen.1007672 (PMC6171964; doi:10.1371/journal.pgen.1007672)
Supplement: S9 Fig — (DOCX) [file pgen.1007672.s028.docx]

Supplementary Figure 9. The distribution of adjusted *p*-values for LFMM given *K*=2 after modifying the genome inflation factor (λ).
