# Supplementary material for: The genomic basis of environmental adaptation in house mice
Source: PLoS Genet. 2018 Sep 24;14(9):e1007672. doi: 10.1371/journal.pgen.1007672 (PMC6171964; doi:10.1371/journal.pgen.1007672)
Supplement: S10 Fig — Distribution of (A) the minimum correlation coefficient (B) the standardized minimum correlation coefficient (C) the minimum slope and (D) the standardized minimum slope for the linear relationship between allele frequencies of SNPs from the exome and latitude. (DOCX) [file pgen.1007672.s029.docx]

Supplementary Figure 10. Distribution of **(A)** the minimum correlation coefficient **(B)** the standardized minimum correlation coefficient **(C)** the minimum slope and **(D)** the standardized minimum slope for the relationship between allele frequencies of SNPs in the exome and latitude. The minimum value for each SNP was determined by comparing results when all populations were included and when any one population was excluded.
