# Supplementary material for: The genomic basis of environmental adaptation in house mice
Source: PLoS Genet. 2018 Sep 24;14(9):e1007672. doi: 10.1371/journal.pgen.1007672 (PMC6171964; doi:10.1371/journal.pgen.1007672)
Supplement: S11 Fig — The distribution of (A) p-values and (B) q-values of the z-scores of the minimum correlation coefficient for SNPs in the exome and the distribution of (C) p-values and (D) q-values of the z-scores of the minimum slope for SNPs in the exome. (DOCX) [file pgen.1007672.s030.docx]

Supplementary Figure 11. The distribution of **(A)** *p*-values and **(B)** *q*-values of the z-scores of the minimum correlation coefficient for SNPs in the exome and the distribution of **(C)** *p*-values and **(D)** *q*-values of the z-scores of the minimum slope for SNPs in the exome.

**B**

**A**

**D**

**C**
