# Supplementary material for: The genomic basis of environmental adaptation in house mice
Source: PLoS Genet. 2018 Sep 24;14(9):e1007672. doi: 10.1371/journal.pgen.1007672 (PMC6171964; doi:10.1371/journal.pgen.1007672)

Supplementary Figure 12. The distribution of average **(A)** R^2^ values and **(B)** average |slope| for 2,500 bp windows in the genome. Blue dashed lines demarcate the mean, grey and red dashed lines demarcate the 95^th^ and 97.5^th^ percentile respectively, and the solid grey line marks the cut-off applied based on the 95^th^ percentile calculated from the average when all populations were included and when any one population was excluded.


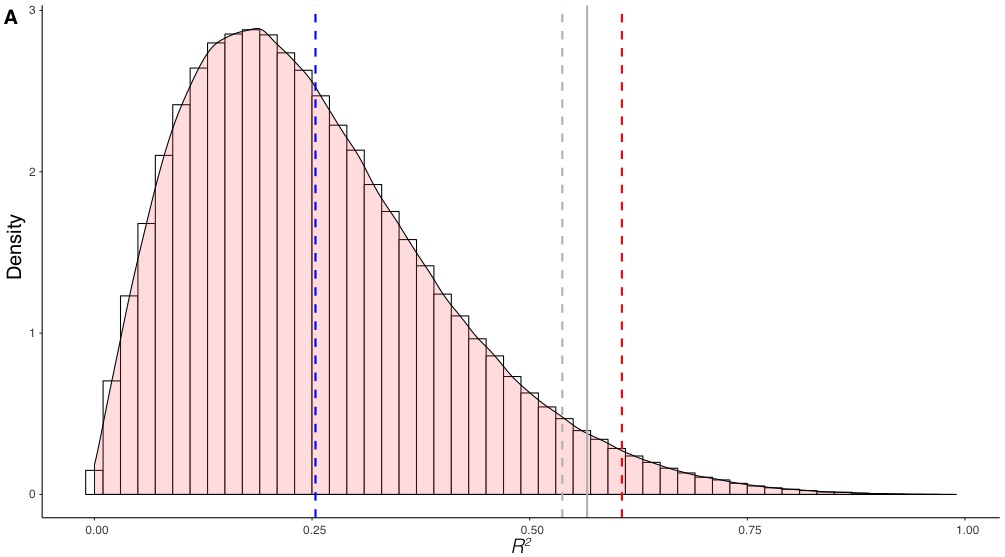


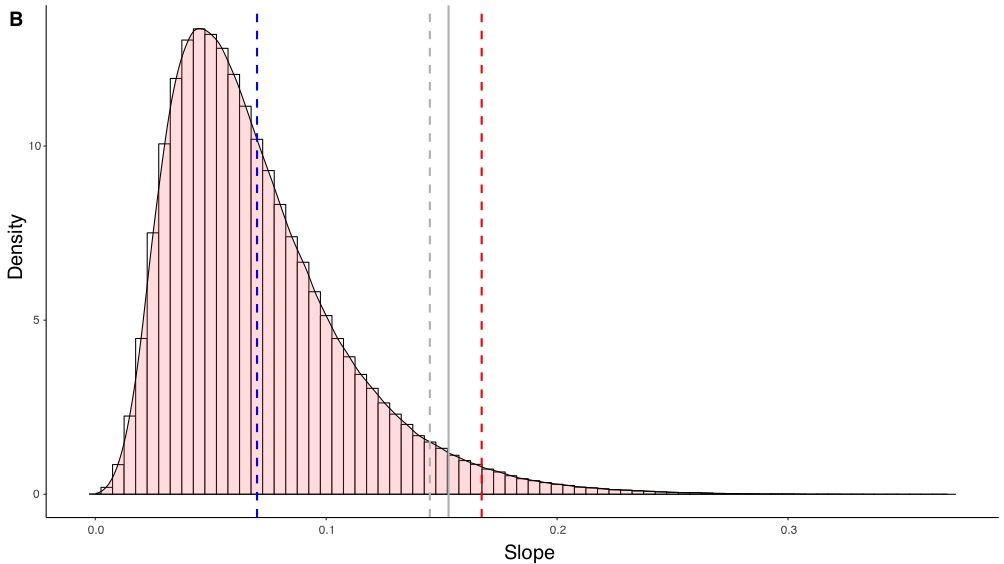

Supplement: S12 Fig — The distribution of (A) average R2 values and (B) average |slope| for 2500 bp windows in the genome. (DOCX) [file pgen.1007672.s031.docx]
