# Supplementary material for: The genomic basis of environmental adaptation in house mice
Source: PLoS Genet. 2018 Sep 24;14(9):e1007672. doi: 10.1371/journal.pgen.1007672 (PMC6171964; doi:10.1371/journal.pgen.1007672)
Supplement: S13 Fig — (DOCX) [file pgen.1007672.s032.docx]

Supplementary Figure 13. Body weight divided by body length for male mice from FL (red) and NY (blue) included in the expression study. Mice that are outliers in terms of expression pattern are noted on the figure.
